# Assessment of contamination, mobility and application of selected technology-critical elements as indicators of anthropogenic pollution of bottom sediments

**DOI:** 10.1007/s11356-024-34377-5

**Published:** 2024-07-30

**Authors:** Magdalena Jabłońska-Czapla, Katarzyna Grygoyć, George Yandem

**Affiliations:** Institute of Environmental Engineering of Polish Academy of Sciences, M. Sklodowskiej-Curie 34 St., 41-819 Zabrze, Poland

**Keywords:** TCE contamination, Spatial and temporal contamination variation, Biała Przemsza River pollution, Mobility of TCE, Emerging contaminants, Indium, Gallium, Germanium

## Abstract

The study investigates the potential of technology-critical elements (TCEs) in the bottom sediments of the Biała Przemsza River as indicators of anthropogenic activities. The mass fractions of TCEs: Ge, Ga, In, Tl, Sb and Te (and other elements) in the sediment were analysed by inductively coupled plasma mass spectrometry with the maximum mass fractions: 2.46, 25.6, 0.528, 27.7, 12.5 and 0.293 mg/kg, respectively. Distribution and identification of TCE sources were supported by statistical analysis (principal component analysis coupled with varimax rotation and hierarchical cluster analysis). Assessments of TCE contamination using the geoaccumulation index, pollution index, contamination factor, enrichment factor and the antimony-to-arsenic ratio highlighted the high contamination of bottom sediments by Sb, Ga, Tl, Cd, As, Zn, Pb and moderate contamination by Co, In and V. Distinct behaviour patterns were observed among TCEs, revealing Sb and Tl as potential indicators of Zn-Pb ore mining activities. Co, V, Ge and, to a lesser extent, Te emerged as promising indicators of coal and coal fly ash effluents. Sequential chemical extraction of TCEs showed that Sb, In and Tl had the highest mobility from sediments. The Risk Assessment Code calculations suggest, that in the Biała Przemsza River bottom sediments, there is an average risk of contamination by As, Tl and Mn. Soluble forms of Tl, Ge, Sb, Te and In were identified in descending order, indicating their bioavailability.

**Supplementary Information:**

The online version contains supplementary material available at 10.1007/s11356-024-34377-5.

## Introduction

Technological development may lead to the release and pollution of the natural environment by technology-critical elements (TCEs) (Cobelo-García et al. 2015; Kouhail et al. 2019, 2022), also known as emerging contaminants (Batley and Campbell 2022). TCEs have become a new potential source of bottom sediment (Bačić et al. 2021; Klein et al. 2022a), water (Filella and Matoušek 2022) and soil (Lucić et al. 2021) contamination. As the global use of TCEs increasingly impacts the environment and the development of new technologies, it is important to better document the concentrations, chemical speciation (or bioavailability), toxicological threshold and capacity for biomagnification of TCEs to assist scientists, policy-makers and industrial and social stakeholders in transitioning to a sustainable economy that is reliant on TCEs (Dang et al. 2021). Technology-critical elements include gallium (Ga), indium (In), germanium (Ge), tellurium (Te), niobium (Nb), antimony (Sb), tantalum (Ta), thallium (Tl), the platinum group elements and most of the rare earth elements (Eurostat. 2023; Filella et al. 2020; Wojcieszek et al. 2018). Among the TCEs, some of the elements belong to the less studied technology-critical elements (LSTCEs) — specifically Ga, In, Ge, Tl and Te — which, together with Sb, are becoming vital to manufacture components for a wide array of advanced and innovative technologies (Jabłońska-Czapla and Grygoyć 2021).

Tl is released into the environment from sulphide ores during their processing and smelting, as well as from fossil fuel combustion. Despite limiting the burning of fossil fuels, study on the Tl concentration in the environment is still very important, due to the toxicity of thallium compounds (Jia et al. 2018). Ge and In accumulations in bottom sediments indicate possible anthropogenic inputs (Klein et al 2022b). Ge has been found in most plants, but its role is not fully understood. It is easily absorbed by plants. In the human body, Ge is concentrated in the kidneys, bones, liver and spleen. It may cause inflammation and mutagenic and teratogenic effects (Tricas et al. 2014). Indium accompanies sulphide metal ore deposits and accumulates in bituminous shales. It also concentrates in coal beds and crude oil (Kabata-Pendias and Pendias 1999). Yang and Chen (2018) showed that In is very toxic to fish species. Ga and In compounds are extensively used in the electro-optical industry; as a consequence, industrial effluents containing Ga and In are discharged into rivers or through irrigation systems and may influence the growth and productivity of crops. Humans may also be exposed to Ga and In via the food chain, which could pose severe health risks (Su et al. 2018). Te is a rare, relatively unknown element with an incompletely understood role in living organisms. It is considered toxic and teratogenic, and there are indications that tellurite, Te(IV), is more toxic than tellurate, Te(VI) (Grandell et al. 2016; Werner et al. 2018). Sb occurs together with deposits of zinc-lead (Zn-Pb) ores, and previous studies have shown the variable nature of Sb concentrations (Jabłońska-Czapla 2015). Sb is released into sediments from various sources: natural (rock composition, weathering of sulphide ores and fluvial processes) and human activities (smelting, metallurgical operations, shooting and application in various technology products like semiconductors to make infrared detectors, diodes and polyethylene terephthalate production) (Bolan et al. 2022; Filella et al. 2002). Exposure to Sb, particularly highly toxic Sb(III) compounds, results in metabolic malfunctioning and impairment of the nervous system and organs (Yang et al. 2015).

Based on prior research, TCEs can be used as indicators of groundwater (Amiel et al. 2021) or surface water (Jabłońska-Czapla and Grygoyć 2022) pollution. The selection of a TCE to indicate environmental pollution should be based on a comprehensive examination of the geogenic substrate of the study area (Jabłońska-Czapla and Grygoyć 2022). Human activity in and around a river basin affects the amount of TCEs in the bottom sediments (Klein et al. 2022b). The bottom sediments of the rivers can absorb metals and metalloids to some degree, serving as indicators of the current conditions within the river ecosystem. It is known that the mobility of elements in bottom sediments is affected by the form in which they occur (Templeton et al. 2000) and the conditions in the river environment. The mobility of many metals increases under acidic conditions (Jabłońska-Czapla et al. 2016).

In Poland, the most industrialised and thus polluted area is Upper Silesia. For decades, human activity conducted in this area has been associated with mining and processing of natural resources in the form of coal or ores. The Biała Przemsza River has an upland character, flowing through the Kraków-Częstochowa Upland and the Silesian Upland. Pollution of this river is caused by both geogenic (Jabłońska-Czapla 2015) and anthropogenic (Ciszewski 2001, 2019) factors. The human activity in this area from the mining and metallurgical industry of Zn-Pb ores and coal mines has increased metal and metalloid pollution in both the water and bottom sediments of this river and thus has had a significant impact on the condition of the surface waters and bottom sediments of this river (Jabłońska-Czapla et al. 2016). The Biała Przemsza River is contaminated with metals (Zn, Pb and Cd), mainly mine water from the Pomorzany Mine, Zn-Pb ores, the Siersza hard coal mine and sewage from the Siersza Power Plant in Olkusz.

This article discusses for the first time the possibility of using several TCEs (Tl, Te, Ga, Ge, In and Sb) as indicators of anthropogenic contamination of bottom sediments using the example of the Biała Przemsza River. In this study, our goal was to investigate the mobility of specific trace critical elements (TCEs) – including Ge, Ga, In, Tl, Sb and Te — in the bottom sediments of the Biała Przemsza River and determine the temporal and spatial variations in their mass fraction. We determined whether the TCEs containing the Biała Przemsza River bottom sediments are mobile and the risk of releasing them into the water under favourable conditions. We examined the mass fraction of elements (cobalt, nickel, copper, uranium, silver, cadmium, chromium, lead, vanadium, arsenic, manganese, zinc, iron and aluminium), physicochemical parameters and granulometry of bottom sediments to specify conditions of the Biała Przemsza River. We determined the degree of TCE contamination using contamination indicators such as the antimony-to-arsenic (Sb/As) ratio, the geoaccumulation index (*I*_*geo*_), the pollution index (*P*_*I*_), the contamination factor (*CF*), enrichment factor (*EF*) and statistical analysis. Distribution and identification of TCE sources were supported by statistical analysis (principal component analysis coupled with varimax rotation and hierarchical cluster analysis).

## Materials and methods

### Study area

**Fig. 1:**
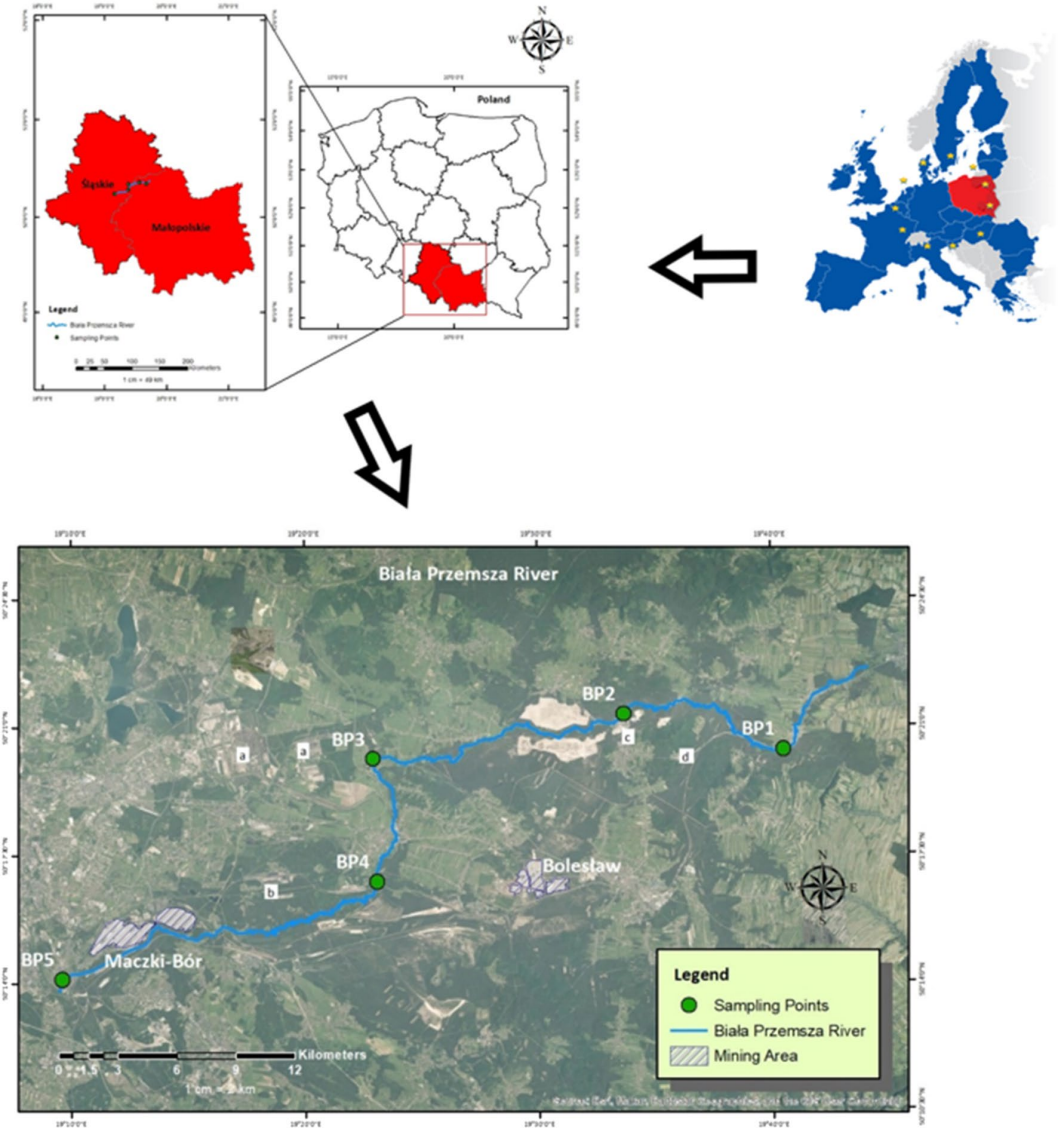
Study area and sampling points: Chrząstowice (BP1), Klucze Osada (BP2), Dąbrowa Górnicza Okradzionów (BP3), Sławków (BP4) and Sosnowiec (BP5)

The Biała Przemsza River (Fig. 1), with a total length of 63.9 km and a basin area of 876.6 km^2^, is a heavily polluted river located in Upper Silesia — the most industrialised and urbanised region in Poland. The river emerges from peat bogs in the Krakowsko-Częstochowska Upland at an altitude of about 376 m and flows through regions of Zn-Pb ore and coal deposits; one of the largest deposits is the Mississippi Valley-type (MVT) in carbonate rocks (Leach et al. 2010; Strzebońska et al. 2017). The Biała Przemsza River enters the Przemsza River — a tributary of one of Poland’s main rivers (the Vistula River) — at an altitude of 242 m. The river’s waters are pulled for municipal use in the upper part of the drainage basin, which has little industrial activity. The water quality declines significantly in the middle and lower reaches of the river due to pollution from a significant amount of wastewater associated with the activities of mining and metallurgical plants, coking plants, steel production and wastewater from power plants. The river also gets wastewater from several nearby municipal wastewater treatment plants, effluents from the drainage of surrounding industrial areas and roads running adjacent to the river and its tributaries. Saline wastewaters from the dewatering of mining pits operated by the zinc Zn-Pb and lead ore mine pits and open-pit sand mines have contributed a large share of the wastewater entering the river. The hydrogeological regime of the catchment area is heavily altered, as a result of the dewatering of mined pits in the surrounding mines, including the open-pit sand mine. The formation of huge depression funnels has contributed to the drying up of many local springs and wetlands. Cessation of mining operations and groundwater pumping in 2020 entailed additional marked changes for the hydrological conditions of the Biała Przemsza River basin, including the drying up of the largest tributaries of the Biała Przemsza River, the Sztoła and Baba Rivers, through which water from the mines had been supplied.

### Field work and sampling

Bottom sediments were sampled at five sites every month from April to October 2021 (Fig. 1). Selection of the sampling sites was preceded by field investigations to characterise sampling locations and to determine accessibility. The points were located along the entire length of the river in its beginning, middle and end zones. Five points were located to safely collect material for testing. The bottom sediments were collected using a grab sampler from the 0–5-cm layer and always from the same sampling points. Figure 1 shows the five sampling points: Chrząstowice (BP1), Klucze Osada (BP2), Dąbrowa Górnicza Okradzionów (BP3), Sławków (BP4) and Sosnowiec (BP5). The geographical locations of the sampling sites are shown in earlier work (Jabłońska-Czapla and Grygoyć 2022). Efforts were made to locate sites so that changes in water quality occurring below natural tributaries and sewage discharge points could be observed. The choice of sampling sites was also influenced by the characteristics of the river. The mountainous nature and fast current made it impossible to collect material for the study.

### Research methodology

#### Physicochemical tests of bottom sediments

Immediately after collecting the bottom sediments in situ, pH, redox potential (Eh) and temperature were measured. Eh was measured with the ERPt-111 electrode (Elmetron, Poland), and pH was measured with the ERH-111 electrode (Elmetron). After air-drying the bottom sediments, a granulometric analysis was carried out in accordance with a standard (PN-EN ISO 17892–4:^2017^–01). Based on the pH and Eh results, the Clark coefficient (Drobnik and Latour 2003) was determined according to the formula:

1 $$\text{rH}=(\text{Eh}+0.068\times \text{pH})/0.03$$

#### Metal analysis in sediments

In the laboratory, samples were averaged, air-dried and sieved through a 2-mm nylon sieve followed by digestion in a Microwave 5000 oven (Anton Paar, Austria) at 190 °C for 50 min (temperature rise over 25 min; temperature hold for 25 min). Bottom sediments were digested in three replicates with spectral purity reagents from Merck (Germany): 5 ml of nitric acid (HNO_3_), 2 ml of hydrogen peroxide (H_2_O_2_) and 3 ml of hydrofluoric acid (HF). Total metal and metalloid (Co, Ni, Cu, In, Ga, U, Ag, Cd, Cr, Pb, V, Sb, As, Mn, Zn, Tl, Te and Ge) contents were determined using inductively coupled plasma mass spectrometry (ICP-MS) with an Elan 6100 DRC-e spectrometer (Perkin Elmer, Waltham, MA, USA) equipped, as described in a previous study (Jabłońska-Czapla and Grygoyć 2022). The iron (Fe) and aluminium (Al) mass fractions were analysed using inductively coupled plasma optical emission spectroscopy (ICP-OES; Avio 200 spectrometer, Perkin Elmer). The TCEs In^115^, Ga^69^, Te^126^, Tl^205^ and Sb^121^ were determined in the standard mode. The correction equation − 0.003404 × Xe129 was used to determine Te. The Ge^74^ content was analysed using a collision chamber with methane as the auxiliary gas, and the correction equation − 0.1385 × Se82 was applied. The concentration of elements was measured three times in each sample.

The following reagents were used for ICP-MS analysis: CAL2-1 multi-element standard solution (AccuStandard®, USA); periodic table mix (Sigma-Aldrich, Switzerland); and metalloid and non-metal mix, antimony standard solution, germanium standard solution and rhodium standard solution (Merck) as internal standards. The following reagents of analytical purity purchased from Chempur (Poland): ammonium sulphate ((NH_4_)_2_SO_4_,), ammonium dihydrogen phosphate ((NH_4_)H_2_PO_4_), diammonium oxalate ((NH_4_)_2_C_2_O_4_ × H_2_O), oxalic acid dihydrate (C_2_H_2_O_4_ × 2H_2_O) and ascorbic acid (C_6_H_8_O_6_); they were used for the sequential extraction procedure. Sequential chemical extraction was carried out in three replicates of bottom sediment samples collected in three seasons: spring (April), summer (July) and autumn (October). All solutions used in this study were prepared in deionised water (5 µS/cm, HLP 10 UV, Hydrolab, Poland).

#### Quality control

The NCS DC 73309 stream sediment certified reference material (China National Analysis Center for Iron and Steel, Beijing, China) was analysed to validate the method used to determine total metals/metalloids and selected TCEs. Table S1 presents the recovery of the tested metals and non-metals in the certified reference material and the basic validation parameters for the determination of elements using ICP-MS. The expanded uncertainty was calculated to define an interval about the result of a measurement that may be expected to encompass a large fraction of the distribution of values that can be reasonably attributed to the measured quantity. The value of the expanded uncertainty of measurement was calculated by the multiplication of the combined measurement uncertainty (*k* = 2), considering the following uncertainty sources: standard measurement in the preparation of the calibration standards, the standard measurement uncertainty of precision, the standard measurement of recovery and the standard measurement of the calibration curve. The value of expanded uncertainty of the method is presented in Table S1.

#### Sequential chemical extraction of the Biała Przemsza River bottom sediments

Among the numerous procedures, Wenzel et al. (2001) optimised one specifically for arsenic, an element that behaves similarly to Sb in soil (Lewińska and Karczewska 2019). Therefore, this approach is most suitable to determine the forms of Sb in soil and bottom sediments. This procedure considers the anionic nature of metalloids and separates them into five fractions: (F1) non-specifically sorbed, which can be considered readily soluble; (F2) specifically sorbed; (F3) associated with amorphous and poorly crystalline Fe and Al hydroxides; (F4) bound to well-crystallised aqueous Fe and Al oxides; and (F5) a residual phase. Fractions F1 and F2 can be considered easily mobilised and therefore potentially dangerous for the environment. The detailed procedure of sequential extraction is presented in Table S2. Fraction F1 is an easily soluble fraction; it includes compounds not specifically bound to the surface of organic matter, hydrated oxides and hydroxides and clays. This form is one of the most mobile: it can be desorbed as a result of changes in the ionic strength of the extraction solutions in contact with the sludge. Fraction F2, named the exchangeable fraction, includes compounds specifically adsorbed on the surface of organic matter particles, hydrated oxides and hydroxides, and clays, as well as incorporated in the structure of carbonate minerals. However, these compounds are not strongly bound and can be desorbed by changing the pH or ionic strength of the solutions. Fraction F3 is associated with amorphous and slightly crystalline hydrated iron oxides, including compounds co-precipitating with iron compounds. Fraction F4 is associated with crystalline, hydrated iron oxides and includes compounds co-precipitating with iron compounds and activated under conditions of reduced redox potential. Fraction F5 is the residual fraction in which metals are embedded in the crystal lattice of the most durable minerals and are unavailable in natural conditions.

Based on the results of sequential chemical extraction, the environmental risk resulting from the form in which TCEs occur in the bottom sediments was assessed. The so-called Risk Assessment Code (RAC) was used. Bottom sediments in which < 1% of metals are associated with ion-exchange, and carbonate fractions are considered safe for the environment. However, sediments in which > 50% of the total metal content is released from the same fractions should be considered highly hazardous, as the released element can easily enter the water and the food chain (Perin et al. 1985). The bioavailability of metals in sediments was considered, and the percentage scale of exchangeable and carbonate fractions was used. The *RAC* value for each analyte is calculated using the following equation:

2 $$RAC = [\text{F}1 +\text{ F}2/(\text{F}1 +\text{ F}2 +\text{ F}3 +\text{F}4 +\text{ F}5)] \times 100{\%}$$

Fractions F1–F5 are the shares of a given element in individual fractions extracted during sequential chemical extraction. The *RAC* results were categorised as follows: < 1%, no risk; 1–10%, low risk; 11–30%, medium risk; 31–50%, high risk; and > 50%, very-high risk.

#### Assessment of bottom sediment pollution in the Biała Przemsza River

*I*_*geo*_, proposed by Müller (1986), is the most widely used indicator for the geoaccumulation of pollutants. It is the binary logarithm of the ratio between the total metal (TM) concentration in the sample divided by the geochemical background concentration of the TM with the correction constant value 1.5, which is used to eliminate the TM fluctuation in the nature and to reduce the anthropogenic effect on the TM concentration in nature. It is calculated from Eq. (3), in which *C*_mSample_ is the TM concentration in the sample; *C*_mBackground_ is the geochemical background concentration of the TM:

3 $${I}_{geo}= {\text{log}}_{2}\left[\frac{{C}_{mSample}}{1.5 \times {C}_{mBackround}}\right]$$

*I*_*geo*_ describes the pollution by an element concerning seven classes from 0 to 6. The class 0 (*I*_*geo*_ ≤ 0) indicates no pollution. The first class (0 < *I*_*geo*_ ≤ 1) describes no contamination or moderate contamination. Second class (1 < *I*_*geo*_ ≤ 2) moderate contamination. The third class (2 < *I*_*geo*_ < 3) concerns moderate or heavy pollution. The heavy contamination is included in the fourth class (3 < *I*_*geo*_ < 4). The fifth class (4 < *I*_*geo*_ < 5) is heavy to very heavy pollution. The last one is the sixth class (*I*_*geo*_ > 5) with the highest contamination.

The *P*_*I*_, shown in Eq. (4), is calculated by dividing *C*_*mSample*_ by *C*_*mBackground*_ for each measurement, while the *CF* is the average of at least five measured samples of TM divided by the background concentration (Dung et al. 2013; Håkanson 1980; Nour et al. 2018).

4 $${P}_{I}= \frac{{C}_{mSample}}{{C}_{mBackground}}$$

The *P*_*I*_ allows one to define the pollution classes of a given bottom sediment as unpolluted (*P*_*I*_ < 1), moderately polluted (1 ≤ *P*_*I*_ ≤ 3) or heavily polluted (*P*_*I*_ > 3). The *CF* (Eq. (5)), which is estimated by the division of the average metal concentration by the background value, is classified into three levels namely: low contamination (*CF* < 1), moderate contamination (1 ≤ *CF* ≤ 3), considerable contamination (3 ≤ *CF* ≤ 6) and very high contamination (*CF* ≥ 6) (Håkanson, 1980).

5 $$CF=\frac{mean({C}_{mSample})}{{C}_{mBackground}}$$

Enrichment factor (*EF*) was determined for all element concentrations to evaluate anthropogenic influences of heavy metals in sediments in the researched area. According to Sutherland (2000), the *EF* is defined as follows:

6 $$EF= \frac{\left(\frac{Me}{Me Ref}\right)Sample }{\left(\frac{Me}{Me Ref}\right)Background}$$

where (*Me/MeRef*)Sample is the metal to reference metal ratio in the samples of interest; (*Me*/*MeRef*)Background is the natural background value of metal to the reference element selected as *MeRef* ratio, also the metal Al was selected as *MeRef* due to its possibility to eliminate the grain-size effect on measured heavy metal concentrations, as has been suggested by Fang et al. (2019). The degree of enrichment is evaluated according to the following criterion: *EF* ≤ 2 indicates deficiency to minimal enrichment; *EF* from 2 to 5 indicates moderate enrichment; *EF* from 5 to 20 indicates significant enrichment; *EF* from 20 to 40 indicates very high enrichment; and *EF* > 40 indicates extremely high enrichment. The background values for typical heavy metals, according to Geochemical Atlas by Lis and Pasieczna (1995), were calculated as the weighted mean of the four locations (Olkusz, Jaworzno, Dąbrowa Górnicza and Mysłowice) where the river passes through. The background values for Te, Sb, Tl and Ga were determined in the same location sampling by Jabłońska-Czapla (2015), while Ge and In were also calculated as the weighted mean by Klein et al. (2022b). Bottom sediment pollution was evaluated based on the Sb/As ratio (Bi et al. 2011; Fu et al. 2011; Sharifi et al. 2016). The size of this ratio can provide information about the source of pollution. The Sb/As ratio used by Jabłońska-Czapla et al. (2023) pointed to the impact of electrowaste from a processing plant on the increase in TCE contamination of soils.

#### Statistical analysis

The mass fractions of the elements were analysed using Statistica 14.0.1 software (TIBCO®). The Shapiro–Wilk test (Shapiro et al. 1968) revealed that the data did not follow a normal distribution. Therefore, the Kruskal–Wallis test followed by post hoc multiple comparisons using the Bonferroni adjustment was used to compare the mass fractions of the elements at BP1–BP5. Moreover, principal component analysis (PCA) coupled with varimax rotation (factorial analysis) (Abdi and Williams 2010) was performed. Spearman’s rank correlation coefficients were calculated to analyse the relationship between the mass fractions of elements and chemical properties. Then, hierarchical cluster analysis (HCA) (Ward 1963) was employed to cluster the correlation matrix coefficients for the elements’ mass fractions only to investigate the behaviour and sources of the observed elements.

## Results and discussion

### Physicochemical parameters and granulometric composition of the Biała Przemsza River bottom sediments

The mass fractions of heavy metals (including TCEs) are sediments influenced by many factors including the sources of the metals, metal mobility, the grain-size fraction of the sediments or physicochemical parameters (Fang et al. 2019). Fig. S3 presents box plots of physicochemical parameter variability of the Biała Przemsza River bottom sediments. Similar to the water (Jabłońska-Czapla and Grygoyć 2022), bottom sediments at BP2 and BP3 had the highest pH, while bottom sediments at BP4 had the lowest pH. The average pH of the bottom sediments was 7.55. This average represents a slight increase compared with a previous study (Jabłońska-Czapla 2015). There have also been noticeable changes in the redox potential of the Biała Przemsza River bottom sediments compared with another previous study (Jabłońska-Czapla et al. 2016). At BP2–BP5, the redox potential was always negative; the redox potential was only positive twice at BP1. This finding indicates that the conditions in this river have improved, as in 2015, positive redox potentials were recorded very often. We measured the highest redox potential (239.8 mV) in April at BP1 and the lowest redox potential (− 349 mV) in October at BP3.

Despite significant differences in the redox potential as well as the concentration of hydrogen ions, the calculated Clark coefficient showed that there are reducing conditions in the Biała Przemsza River bottom sediments; the Clark coefficient was 9. On the Clark scale (from 3 to 23), the values reducing conditions occur at rH < 15 while oxidising conditions occur at rH > 25 (Drobnik and Latour 2003). Only the bottom sediments collected in April from BP1 had a Clark coefficient of 23, which indicates oxidising conditions (Table S5).

We tested the Biała Przemsza River bottom sediments to determine the granulometric composition during three seasons of the year: spring, summer and autumn. (Table S6). Irrespective of the season, at BP1, BP2, BP4 and BP5, the bottom sediments consisted mainly of medium sand fractions with a grain diameter between 0.5 and 0.2 mm. Only the bottom sediments from BP3 consisted mainly of the finest granulation fraction (< 0.01 mm) — that is, the dust and clay fraction. In the bottom sediments, the share of fractions with granulation of 0.2–0.1 mm (fine sand) increased along the course of the river from BP1 to BP4; it was the highest (38.6%) at BP4, almost equalling the percentage share of the 0.5–0.2 mm fraction, which was 41.8%.

### Distribution of selected TCEs in the Biała Przemsza River bottom sediments

Discussing the TCE mass fractions in bottom sediments and relating these mass fractions to reference values are difficult, because neither the detailed geochemical map of Upper Silesia (2021) nor the geochemical atlas of Europe (Salminen et al. 2005) contains information on the tested TCEs. The only approach is to compare the obtained results with those obtained by other researchers. Table 1 shows the metal and metalloid contents of the Biała Przemsza River bottom sediments.

**Table 1:** Metal(loid)s mass fractions (< 2 mm) in the bottom sediments of the Biała Przemsza River at various sampling points: BP1 — Chrząstowice; BP 2 — Klucze Osada; BP 3 — Dąbrowa Górnicza Okradzionów; BP 4 — Sławków; and BP 5 — Sosnowiec

Date	Point	Te	In	U	Ge	Ag	Sb	Tl	Co	Ga
		mg/kg								
April	**BP1**	< 0.02	< 0.05	0.286 ± 0.035	1.15 ± 0.18	0.120 ± 0.017	0.155 ± 0.022	0.109 ± 0.017	1.2 ± 0.16	6.91 ± 0.71
	**BP2**	< 0.02	< 0.05	0.505 ± 0.062	1.00 ± 0.16	0.496 ± 0.072	0.585 ± 0.082	0.225 ± 0.035	2.41 ± 0.33	9.38 ± 0.97
	**BP3**	0.070 ± 0.009	< 0.05	0.89 ± 0.11	1.17 ± 0.18	1.15 ± 0.17	1.54 ± 0.22	4.29 ± 0.67	10.4 ± 1.4	16.2 ± 1.7
	**BP4**	0.038 ± 0.005	1.0 ± 0.12	0.89 ± 0.11	1.16 ± 0.18	1.59 ± 0.23	2.38 ± 0.33	4.22 ± 0.66	4.46 ± 0.61	17.8 ± 1.8
	**BP5**	0.081 ± 0.011	< 0.05	0.726 ± 0.089	1.46 ± 0.23	1.22 ± 0.18	1.47 ± 0.21	5.21 ± 0.82	8.0 ± 1.1	7.74 ± 0.79
May	**BP1**	< 0.02	< 0.05	0.309 ± 0.038	0.99 ± 0.15	< 0.1	0.193 ± 0.027	0.099 ± 0.016	0.494 ± 0.067	3.30 ± 0.34
	**BP2**	< 0.02	< 0.05	0.560 ± 0.069	1.11 ± 0.17	0.287 ± 0.42	0.674 ± 0.095	0.177 ± 0.028	2.02 ± 0.27	7.23 ± 0.74
	**BP3**	0.143 ± 0.019	0.476 ± 0.062	2.02 ± 0.25	1.64 ± 0.25	5.03 ± 0.72	9.0 ± 1.3	15.7 ± 2.5	8.0 ± 1.1	15.9 ± 1.6
	**BP4**	0.065 ± 0.09	0.151 ± 0.02	0.87 ± 0.11	1.48 ± 0.23	1.79 ± 0.26	2.62 ± 0.39	4.32 ± 0.68	3.73 ± 0.51	10.1 ± 1.0
	**BP5**	0.240 ± 0.032	0.15 ± 0.02	1.45 ± 0.18	2.10 ± 0.33	3.26 ± 0.47	4.66 ± 0.66	7.4 ± 1.2	17.1 ± 2.3	18.5 ± 1.9
June	**BP1**	< 0.02	< 0.05	0.352 ± 0.043	1.32 ± 0.20	0.152 ± 0.022	0.263 ± 0.037	0.183 ± 0.029	1.23 ± 0.17	6.21 ± 0.64
	**BP2**	< 0.02	< 0.05	0.672 ± 0.083	1.05 ± 0.16	0.513 ± 0.074	1.04 ± 0.15	0.25 ± 0.40	2.73 ± 0.37	9.8 ± 1.0
	**BP3**	0.182 ± 0.024	0.213 ± 0.028	2.46 ± 0.30	1.67 ± 0.26	5.34 ± 0.77	8.9 ± 1.2	17.2 ± 2.7	10.8 ± 1.5	17.3 ± 1.78
	**BP4**	0.05 ± 0.007	0.114 ± 0.015	0.99 ± 0.12	1.27 ± 0.2	1.76 ± 0.25	2.97 ± 0.42	4.98 ± 0.78	5.18 ± 0.70	15.2 ± 1.6
	**BP5**	0.173 ± 0.023	0.121 ± 0.016	1.29 ± 0.16	1.98 ± 0.31	2.63 ± 0.38	3.78 ± 0.53	8.3 ± 1.3	11.3 ± 1.5	17.3 ± 1.8
July	**BP1**	0.0225 ± 0.003	< 0.05	0.373 ± 0.046	1.13 ± 0.18	0.193 ± 0.028	0.524 ± 0.074	0.199 ± 0.031	1.14 ± 0.15	5.27 ± 0.54
	**BP2**	0.041 ± 0.005	< 0.05	1.04 ± 0.13	1.12 ± 0.17	0.486 ± 0.070	1.07 ± 0.15	0.290 ± 0.045	2.71 ± 0.37	9.47 ± 0.97
	**BP3**	0.286 ± 0.038	0.316 ± 0.041	3.26 ± 0.40	2.35 ± 0.37	8.4 ± 1.2	11.18 ± 1.6	27.7 ± 4.3	11.0 ± 1.5	18.3 ± 1.9
	**BP4**	0.056 ± 0.007	0.087 ± 0.011	1.00 ± 0.12	1.28 ± 0.2	1.95 ± 0.28	2.44 ± 0.34	5.23 ± 0.82	5.01 ± 0.68	12.1 ± 1.2
	**BP5**	0.293 ± 0.039	0.129 ± 0.017	1.95 ± 0.24	2.46 ± 0.38	3.40 ± 0.49	5.26 ± 0.74	9.2 ± 1.4	19.2 ± 2.6	21.0 ± 2.2
August	**BP1**	0.039 ± 0.005	< 0.05	0.359 ± 0.044	1.24 ± 0.19	0.192 ± 0.028	0.314 ± 0.044	0.170 ± 0.027	0.746 ± 0.10	5.11 ± 0.53
	**BP2**	0.049 ± 0.006	< 0.05	0.94 ± 0.12	1.15 ± 0.18	0.487 ± 0.071	1.22 ± 0.17	0.278 ± 0.044	2.67 ± 0.36	8.74 ± 0.90
	**BP3**	0.208 ± 0.028	0.438 ± 0.057	2.78 ± 0.34	2.0 ± 0.32	8.1 ± 1.2	11.4 ± 1.6	22.0 ± 3.5	8.90 ± 1.2	20.0 ± 2.1
	**BP4**	0.042 ± 0.006	< 0.05	0.98 ± 0.12	1.36 ± 0.21	1.63 ± 0.24	1.85 ± 0.26	4.54 ± 0.71	4.58 ± 0.62	11.6 ± 1.2
	**BP5**	0.102 ± 0.013	< 0.05	0.82 ± 0.10	1.54 ± 0.24	0.98 ± 0.14	1.45 ± 0.20	2.97 ± 0.47	7.8 ± 1.1	12.2 ± 1.3
September	**BP1**	0.028 ± 0.004	< 0.05	0.385 ± 0.047	0.937 ± 0.15	0.163 ± 0.024	0.329 ± 0.046	0.131 ± 0.021	1.24 ± 0.17	8.85 ± 0.91
	**BP2**	0.034 ± 0.004	< 0.05	0.634 ± 0.078	1.18 ± 0.18	0.400 ± 0.058	0.78 ± 0.11	0.184 ± 0.029	2.86 ± 0.39	10.0 ± 1.0
	**BP3**	0.11 ± 0.015	0.243 ± 0.032	2.25 ± 0.227	1.55 ± 0.24	5.50 ± 0.80	7.8 ± 1.1	14.4 ± 2.3	12.7 ± 1.7	25.6 ± 2.6
	**BP4**	0.032 ± 0.004	< 0.05	0.565 ± 0.069	1.30 ± 0.20	0.73 ± 0.11	0.533 ± 0.075	1.48 ± 0.23	2.12 ± 0.29	4.29 ± 0.44
	**BP5**	0.076 ± 0.010	< 0.05	0.91 ± 0.11	1.43 ± 0.22	0.88 ± 0.13	1.30 ± 0.18	2.51 ± 0.39	5.99 ± 0.81	8.08 ± 0.83
October	**BP1**	0.028 ± 0.004	< 0.05	0.243 ± 0.030	1.08 ± 0.17	0.095 ± 0.014	0.232 ± 0.033	0.086 ± 0.013	0.83 ± 0.11	6.09 ± 0.63
	**BP2**	< 0.02	< 0.05	0.451 ± 0.055	< 0.2	0.226 ± 0.033	0.436 ± 0.061	0.147 ± 0.023	2.03 ± 0.28	8.92 ± 0.92
	**BP3**	0.171 ± 0.023	0.528 ± 0.069	2.40 ± 0.29	1.78 ± 0.28	9.5 ± 1.4	12.5 ± 1.8	23.2 ± 3.6	9.2 ± 1.2	13.8 ± 1.4
	**BP4**	0.035 ± 0.005	< 0.05	0.614 ± 0.075	1.11 ± 0.17	0.72 ± 0.10	0.62 ± 0.25	1.42 ± 0.22	2.56 ± 0.35	7.14 ± 0.74
	**BP5**	0.220 ± 0.029	0.101 ± 0.013	2.00 ± 0.25	2.13 ± 0.33	2.76 ± 0.40	4.64 ± 0.65	6.99 ± 1.1	16.9 ± 2.3	15.8 ± 1.6

Date	V	Ni	Cr	Cu	Cd	As	Mn	Pb	Zn	Al	Fe
								g/kg			
April	3.76 ± 0.32	1.63 ± 0.23	11.1 ± 1.9	2.31 ± 0.30	0.092 ± 0.009	0.366 ± 0.060	180 ± 25	0.008 ± 0.001	0.016 ± 0.004	4.57 ± 0.96	1.11 ± 0.19
	9.36 ± 0.79	4.9 ± 0.7	27.4 ± 4.7	8.1 ± 1.0	1.58 ± 0.15	1.45 ± 0.24	150 ± 20	0.057 ± 0.006	0.171 ± 0.040	4.66 ± 0.98	4.06 ± 0.70
	9.70 ± 0.82	13.2 ± 1.9	29.3 ± 5.1	15.1 ± 1.9	37.8 ± 3.6	37.2 ± 6.1	940 ± 130	1.33 ± 0.13	3.63 ± 0.86	2.60 ± 0.55	7.7 ± 1.3
	7.62 ± 0.64	7.2 ± 1.0	11.7 ± 2.0	27.1 ± 3.5	44.5 ± 4.3	60.0 ± 9.8	270 ± 36	1.68 ± 0.16	3.92 ± 0.92	1.18 ± 0.25	9.0 ± 1.6
	8.94 ± 0.76	11.2 ± 1.6	15.5 ± 2.7	14.2 ± 1.8	34.1 ± 3.3	32.9 ± 5.4	870 ± 120	1.31 ± 0.13	3.63 ± 0.86	2.52 ± 0.53	8.4 ± 1.5
May	1.84 ± 0.16	0.676 ± 0.096	8.1 ± 1.4	0.96 ± 0.12	0.104 ± 0.010	0.312 ± 0.051	240 ± 32	0.007 ± 0.001	0.022 ± 0.005	3.18 ± 0.67	1.00 ± 0.17
	7.01 ± 0.59	3.22 ± 0.46	19.8 ± 3.4	10.2 ± 1.3	1.24 ± 0.12	1.36 ± 0.22	140 ± 19	0.057 ± 0.006	0.120 ± 0.028	4.86 ± 1.0	4.36 ± 0.75
	15.5 ± 1.3	17.8 ± 2.5	16.5 ± 2.9	130 ± 17	110 ± 10	230 ± 37	780 ± 100	5.31 ± 0.52	10.2 ± 2.4	0.94 ± 0.20	19.6 ± 3.4
	6.46 ± 0.55	6.99 ± 0.99	7.56 ± 1.3	31.4 ± 4.0	50.3 ± 4.8	61.9 ± 10	234 ± 31	1.72 ± 0.17	3.79 ± 0.89	0.70 ± 0.15	8.7 ± 1.5
	32.7 ± 2.8	36.0 ± 5.1	48.2 ± 8.3	79 ± 10	96.0 ± 9.2	73.9 ± 12	1880 ± 250	1.97 ± 0.19	6.0 ± 1.4	2.35 ± 0.50	28.5 ± 4.9
June	3.65 ± 0.31	1.96 ± 0.28	15.3 ± 2.6	2.05 ± 0.26	0.351 ± 0.034	0.861 ± 0.14	173 ± 23	0.016 ± 0.002	0.029 ± 0.007	3.87 ± 0.82	1.85 ± 0.32
	10.1 ± 0.85	4.87 ± 0.69	33.2 ± 5.7	4.89 ± 0.63	1.98 ± 0.19	2.71 ± 0.44	169 ± 23	0.079 ± 0.008	0.193 ± 0.045	4.47 ± 0.94	3.75 ± 0.65
	15.0 ± 1.27	27.5 ± 3.9	19.1 ± 3.3	90 ± 11	89.2 ± 8.6	300 ± 49	970 ± 130	5.62 ± 0.55	10.0 ± 2.3	1.23 ± 0.26	26.8 ± 4.6
	8.22 ± 0.69	8.16 ± 1.2	10.7 ± 1.8	28.0 ± 3.6	56.3 ± 5.4	69.2 ± 11	310 ± 41	1.89 ± 0.18	4.3 ± 1.0	0.96 ± 0.20	9.6 ± 1.7
	29.3 ± 2.5	29.2 ± 4.1	40.2 ± 7.0	56.1 ± 7.2	76.3 ± 7.3	57.2 ± 9.3	1200 ± 160	1.72 ± 0.17	5.2 ± 1.2	2.56 ± 0.54	28.7 ± 5.0
July	3.50 ± 0.30	2.81 ± 0.40	18.8 ± 3.2	1.80 ± 0.23	0.507 ± 0.049	0.97 ± 0.16	150 ± 19	0.019 ± 0.002	0.069 ± 0.016	5.6 ± 1.2	3.12 ± 0.54
	11.7 ± 0.99	6.39 ± 0.91	42.5 ± 7.3	7.44 ± 0.95	2.24 ± 0.22	2.24 ± 0.36	250 ± 34	0.088 ± 0.009	0.219 ± 0.052	4.65 ± 0.98	7.0 ± 1.2
	27.6 ± 2.3	32.3 ± 4.6	24.6 ± 4.2	140 ± 18	148 ± 14	435 ± 71	1100 ± 150	7.99 ± 0.78	17.100 ± 4.0	1.68 ± 0.35	30.0 ± 5.2
	9.15 ± 0.77	9.26 ± 1.3	10.5 ± 1.8	27.9 ± 3.6	65.0 ± 6.3	80.4 ± 13	360 ± 48	1.95 ± 0.190	5.1 ± 1.2	1.05 ± 0.22	10.9 ± 1.9
	32.4 ± 2.7	36.7 ± 5.2	45.0 ± 7.8	80 ± 10	112 ± 11	71 ± 12	1800 ± 260	2.26 ± 0.22	6.3 ± 1.5	2.39 ± 0.504	29.4 ± 5.1
August	2.97 ± 0.25	1.79 ± 0.25	11.2 ± 1.9	0.433 ± 0.055	0.206 ± 0.020	0.607 ± 0.099	100 ± 14	0.011 ± 0.001	0.042 ± 0.010	6.6 ± 1.4	2.05 ± 0.35
	12.8 ± 1.1	6.63 ± 0.94	43 ± 7.4	7.9 ± 1.0	2.89 ± 0.28	3.27 ± 0.53	220 ± 29	0.098 ± 0.010	0.232 ± 0.055	4.8 ± 1.0	7.4 ± 1.3
	25.2 ± 2.1	29.3 ± 4.1	27.2 ± 4.7	140 ± 18	175 ± 17	342 ± 56	1000 ± 130	7.90 ± 0.77	16.0 ± 3.8	1.45 ± 0.31	25.3 ± 4.4
	7.99 ± 0.67	8.5 ± 1.2	10.0 ± 1.7	16.4 ± 2.1	81.3 ± 7.8	67 ± 11	312 ± 42	1.26 ± 0.12	5.8 ± 1.4	0.81 ± 0.17	10.2 ± 1.8
	11.5 ± 0.97	12.9 ± 1.8	17.5 ± 3.0	18.1 ± 2.3	28.0 ± 2.7	14.8 ± 2.4	890 ± 120	0.50 ± 0.048	1.96 ± 0.46	5.6 ± 1.2	11.0 ± 1.9
September	3.94 ± 0.33	2.02 ± 0.29	18.5 ± 3.2	3.01 ± 0.38	0.258 ± 0.025	0.856 ± 0.14	120 ± 16	0.012 ± 0.001	0.046 ± 0.011	7.0 ± 1.5	2.17 ± 0.37
	9.29 ± 0.78	5.57 ± 0.79	31.8 ± 5.5	6.95 ± 0.89	1.59 ± 0.15	1.81 ± 0.29	130 ± 18	0.062 ± 0.006	0.138 ± 0.032	5.5 ± 1.2	5.39 ± 0.93
	20.0 ± 1.7	21.5 ± 3.0	19.7 ± 3.4	150 ± 19	267 ± 26	290 ± 47	910 ± 120	7.30 ± 0.72	14.3 ± 3.4	1.83 ± 0.39	21.6 ± 3.7
	3.86 ± 0.33	3.45 ± 0.49	6.3 ± 1.1	4.53 ± 0.58	18.8 ± 1.8	12.9 ± 2.1	160 ± 21	0.483 ± 0.047	2.31 ± 0.54	3.00 ± 0.63	4.48 ± 0.77
	11.40 ± 0.96	11.3 ± 1.6	19.1 ± 3.3	21.0 ± 2.7	35.4 ± 3.4	11.5 ± 1.9	580 ± 77	0.345 ± 0.034	1.98 ± 0.47	5.8 ± 1.2	10.0 ± 1.7
October	1.82 ± 0.15	1.20 ± 0.17	7.5 ± 1.3	1.61 ± 0.21	0.163 ± 0.016	0.605 ± 0.099	170 ± 23	0.008 ± 0.001	0.027 ± 0.006	4.44 ± 0.94	1.28 ± 0.22
	6.18 ± 0.52	3.05 ± 0.43	15.5 ± 2.7	3.43 ± 0.44	0.787 ± 0.076	1.36 ± 0.22	77 ± 10	0.037 ± 0.004	0.070 ± 0.016	6.7 ± 1.4	2.73 ± 0.47
	27.0 ± 2.3	29.8 ± 4.2	22.2 ± 3.8	210 ± 26	370 ± 36	390 ± 64	1100 ± 150	9.35 ± 0.91	17.40 ± 0.41	1.56 ± 0.33	27.8 ± 4.8
	4.57 ± 0.39	4.35 ± 0.62	7.33 ± 1.3	6.32 ± 0.81	17.0 ± 1.6	15.4 ± 2.5	100 ± 14	0.567 ± 0.055	1.83 ± 0.43	3.61 ± 0.76	4.17 ± 0.72
	32.1 ± 2.7	31.9 ± 4.5	49.4 ± 8.5	64.5 ± 8.2	170 ± 16	44.1 ± 7.2	1720 ± 230	1.15 ± 0.11	6.1 ± 1.4	3.84 ± 0.81	25.7 ± 4.5

Among the tested TCEs (Ga, In, Sb, Te, Tl and Ge), we found the highest mass fraction of Ga at BP3 (25.6 mg/kg). The average Ga mass fraction in the Biała Przemsza River bottom sediments was 11.8 mg/kg. As shown in previous work (Jabłońska-Czapla and Grygoyć 2022), at Dąbrowa Górnicza Okradzionów, Ga, Tl and Sb could originate naturally from MVT deposits and anthropogenically from a century of mining activity in MVT deposit areas. The occurrence of Ga in the Silesia-Cracow area ranges from 1.50 to 177 mg/kg (Mikulski et al. 2018). For comparison, the average Ga mass fraction in the Rhine River (Klein et al. 2022b) was 12.3 mg/kg and 14.0–23.0 mg/kg in the North Sea bottom sediments (Klein et al. 2022a), while the mass fraction of this element in the Biała Przemsza River was 25.6 mg/kg. The Ga mass fraction increased in the bottom sediments along the course of the river, with the highest mass fraction occurring once at BP3 and once at BP5.

Similar to Ga, there was a high Te mass fraction at BP3. The highest Te mass fraction occurred at BP3 and BP5, specifically 0.293 mg/kg in July. The Te mass fraction increased from April to July and then decreased from July to October. In the Biała Przemsza River bottom sediments, the mass fraction of this element was ten times higher compared to the content of this element in the bottom sediments of Croatian lakes (Bačić et al. 2021) or surface sediments collected from the Changjiang Estuary (Duan et al. 2014). German researchers obtained similar results: the Te mass fraction in the Rhine River bottoms sediments ranged from 0.12 to 0.26 mg/kg (Klein et al. 2022b).

**Fig. 2:**
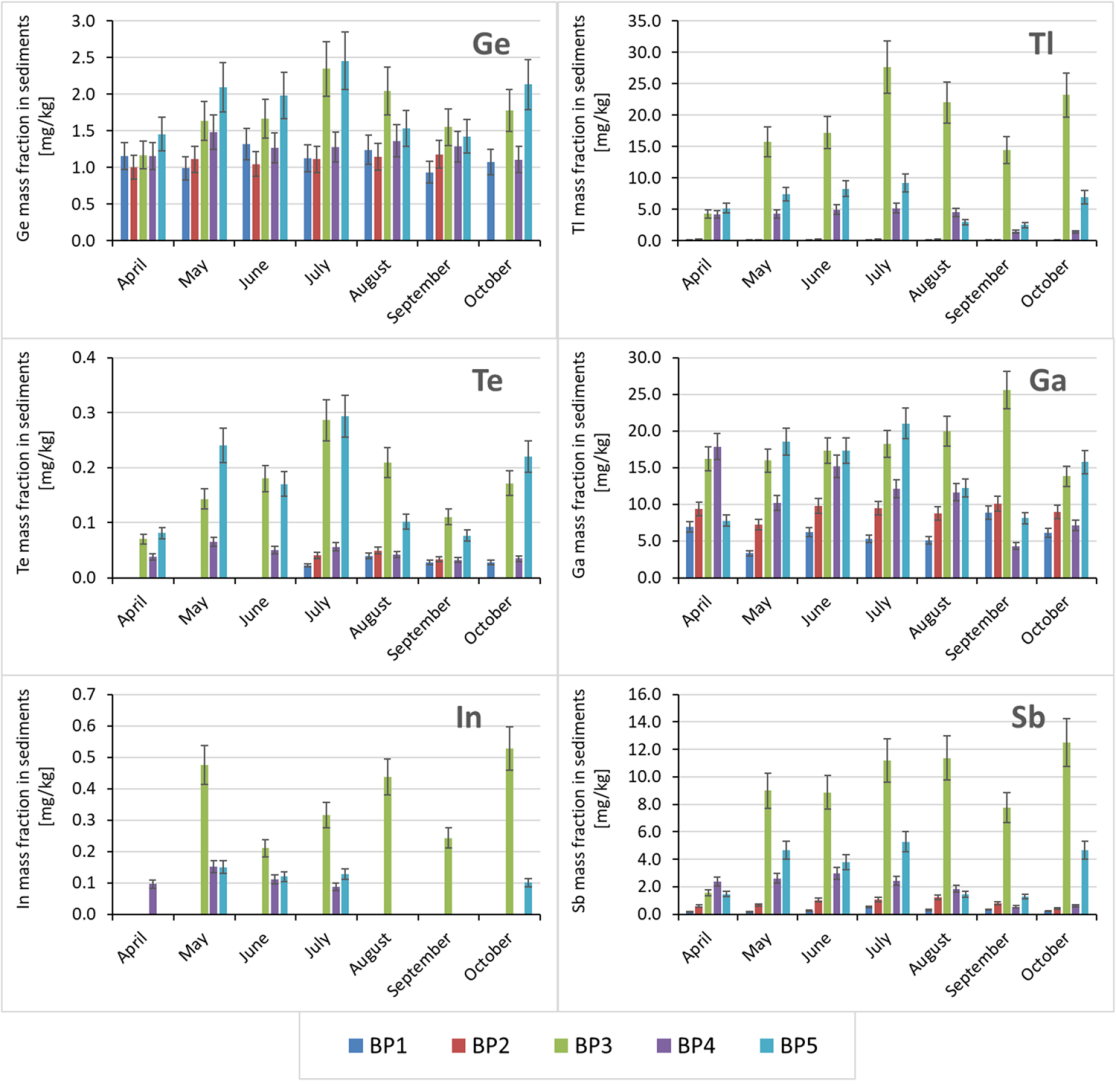
Temporal and spatial variability in the mass fraction (< 2 mm) of selected technology-critical elements (TCEs) in the bottom sediments of the Biała Przemsza River at various sampling points: BP1 — Chrząstowice, BP2 — Klucze; BP3 — Dąbrowa Górnicza Okradzionów; BP4 — Sławków; and BP5 — Sosnowiec. The ranges of error bars were adjusted according to the uncertainties during measurements

**Fig. 3:**
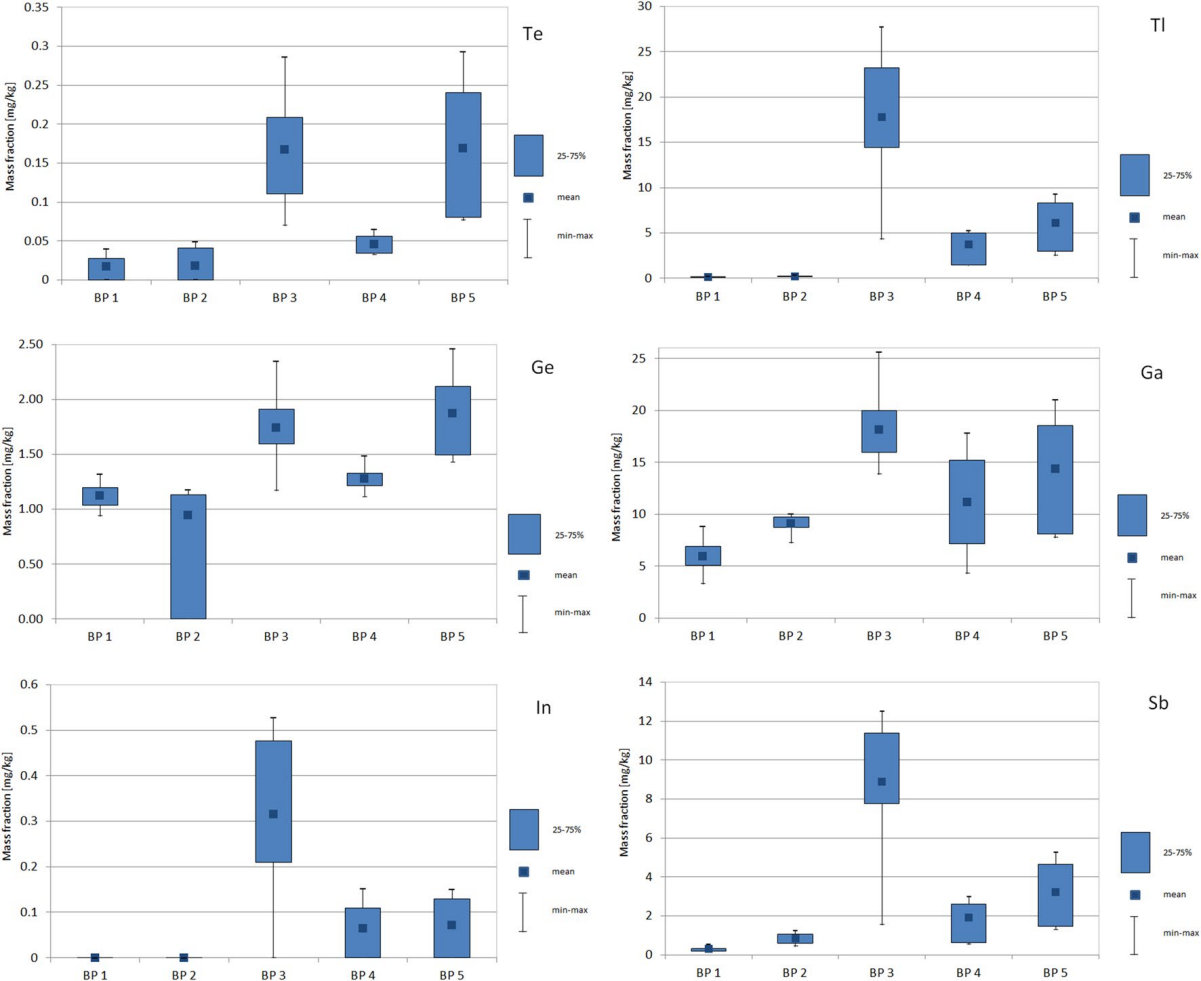
Box plot showing the variability in the mass fraction (< 2 mm) of selected technology-critical elements (TCEs) in the Biała Przemsza bottom sediments at different sampling points: BP1 — Chrząstowice, BP2 — Klucze; BP3 — Dąbrowa Górnicza Okradzionów; BP4 — Sławków; and BP5 — Sosnowiec

The Biała Przemsza River flows through areas rich in Zn-Pb ore deposits. Tl is bound to Zn-Pb ores, which occurred in significant amounts at BP3–BP5. The Tl mass fraction in the soils in this area range from 0.14 to 110 mg/kg (Mikulski et al. 2018). As presented in Figs. 2 and 3, the Tl mass fraction in the Biała Przemsza River bottom sediments shows great variability, increases from April to July and then decreases in autumn. We observed the highest Tl mass fraction at each sampling time at BP3 (27.7 mg/kg) and the lowest Tl mass fraction at BP1 and BP2 (below 1 mg/kg). There was a similar trend in the Tl mass fraction at BP4 and BP5. Tl and many of its compounds, especially sulphates, have been classified as very toxic. High mass fraction of these compounds in the environment is particularly dangerous for living organisms. The Biała Przemsza River bottom sediments were significantly contaminated with Tl. The maximum Tl mass fraction in the bottom sediments occurred in June and was > 25.0 mg/kg. For comparison, the average Tl mass fraction in the bottom sediments collected from Chesapeake Bay, Baltimore, MD, USA, was 0.84 mg/kg (Dolor et al. 2009). The Tl mass fraction in the bottom sediments of other Polish rivers is equally low. For example, in the Kłodnica River, which flows through the central part of the Upper Silesian metropolis, the maximum Tl mass fraction in the bottom sediments was 1.17 mg/kg (Jabłońska-Czapla and Grygoyć 2023). Extremely high Tl mass fraction in the Biała Przemsza River bottom sediments have a geogenic origin. Due to its considerable Tl pollution, the Biała Przemsza River poses an ecological threat to living organisms.

The Ge mass fraction in the bottom sediments also showed marked variability. Germanium mass fraction increased along the course of the river with the highest mass fraction at BP3 and BP5. The Ge mass fraction in the bottom sediments was 0.937–2.46 mg/kg, with the maximum Ge mass fraction which occurred in July at BP5 and was 2.46 mg/kg. As shown in Fig. 2, in most cases the Ge mass fraction in the bottom sediments is similar, that is, between 1.0 and 1.5 mg/kg. Only in a few cases (at BP3 and BP5) Ge mass fraction in the bottom sediments exceed 2.0 mg/kg.

Previous research has indicated that the mass fraction of germanium in the suspended solids of the Biała Przemsza River rises as the river flows downstream, reaching a peak value of 2.5 mg/kg (Jabłońska-Czapla and Grygoyć, 2022). Croatian scientists obtained similar results when examining soil samples taken along the Sava River flowing through Slovenia and Croatia (Lucić et al. 2021). The average Ge mass fraction in these samples was 1.1 mg/kg, and the highest Ge mass fraction in these soils was 2.3 mg/kg. Furthermore similar Ge mass fraction was found in the middle of the Rhine River (Klein et al. 2022b). Klein et al. (2022b) reported an increase in the Ge mass fraction in the bottom sediments along the course of the Rhine River and pointed to the anthropogenic nature of Ge pollution of the bottom sediments. The same authors obtained similar results when examining bottom sediments collected from the North Sea (Klein et al. 2022a). The Ge mass fraction ranged from 1.4 to 2.3 mg/kg in the bottom sediments in the north and from 1.2 to 1.9 mg/kg in the bottom sediments collected in the south of this sea. The Ge mass fraction in the bottom sediments of the Chesapeake Bay near Baltimore, MD, USA, averaged 1.9–2.2 mg/kg (Dolor et al. 2009).

Similar to Tl, In was present in the Biała Przemsza River bottom sediments, mainly at BP3–BP5 points. The maximum In mass fraction occurred at BP3 (0.528 mg/kg). The In mass fraction in the Biała Przemsza River bottom sediments was often very low (below 0.05 mg/kg). Figure 2 shows that similar to Sb and Tl, there is great variability in the In mass fraction. The results indicate slight In contamination of bottom sediments. Klein et al. (2022b) studied the bottom sediments of the Rhine River and found that the In mass fraction was 0.054 mg/kg in the northern part of the river, 0.082 mg/kg in the middle reaches of the river and 0.090 mg/kg In in the lower reaches of the river. Thus, there is greater anthropogenic In pollution of the Rhine River. The bottom sediments of the Chesapeake Bay near Baltimore, MD, USA, were also not contaminated with In, with an average mass fraction of 0.33 mg/kg. The authors examined the cores of bottom sediments and indicated the anthropogenic nature of the pollution, characterized by the enrichment of individual sediment layers with elements such as In, Ge, Ga and Nb (Dolor et al. 2009). In areas contaminated with metals such as Cd, Zn and Pb, the In mass fraction was 0.107–1.92 mg/kg, while in natural soils the In mass fraction was 0.02–0.08 mg/kg (Asami et al. 1990; Rudnick and Gao 2003; Wedepohl 1995).

Among the tested TCEs, the Sb mass fraction, which was evaluated in this river in 2015, also increased. The Sb mass fraction increased significantly at BP3, with a maximum of 12.5 mg/kg. Similarly, the mass fraction of arsenic in bottom sediments is high, reaching a maximum mass fraction at the same sampling point of almost 435 mg/kg, which is higher compared to other Silesian rivers (Jabłońska-Czapla et al. 2020; Jabłońska-Czapla and Grygoyć 2023).

The sediments of the Biała Przemsza River were heavily polluted with heavy metals, of which the Pb and Zn mass fraction classify this river as class III according to the geochemical criteria (Bojakowska and Sokołowska 1998). It should be noted that the high mass fraction of Zn and Pb and the accompanying Sb or Cd have a geogenic origin, as the Biała Przemsza River flows through areas rich in Zn-Pb ores of the Silesia-Cracow area (Mikulski et al. 2018). Mining of Zn-Pb ores in the Biała Przemsza catchment began in the sixteenth century, and large-scale mining operations were carried out from the mid-twentieth century. The Zn-Pb ore deposits of the Upper Silesian-Cracow area are similar to the MVT type (Leach et al. 2010). In these ores, there are also increased mass fraction of TCEs such as Ga, hafnium (Hf) and Tl.

Among the less frequently studied elements, we can mention about uranium. The uranium mass fraction in the Biała Przemsza River bottom sediments was the highest at the third sampling point and amounted to 3.26 mg/kg. However, the average uranium mass fraction does not differ significantly from that measured in another Polish river, namely the Kłodnica River (Jabłońska-Czapla and Grygoyć 2023). Compared to other rivers, especially those flowing through uranium-rich areas (Dong et al. 2021), the uranium mass fraction in the Biała Przemsza River sediments does not exceed the geochemical background for this region.

#### Statistical analysis and enrichment of bottom sediments

When comparing the mass fractions of different elements, it was found that TCEs had consistently lower means and standard deviations at all sampling points (Table S3) compared to Cd, As, Mn, Pb and Zn. This indicates that the mass fractions of TCEs did not fluctuate significantly. On the other hand, the mass fractions of Cd, As, Mn, Pb and Zn showed significant spikes at BP3, BP4 and BP5 sampling locations, as evidenced by their high standard deviations (Table 1 and Table S3).

Based on the analysis of the correlation matrix (Table S4) between the parameters, we can conclude that the TCE mass fraction in the Biała Przemsza River bottom sediments correlate with each other. There are strong correlations between TCEs and Pb, Cu and Zn (Fig. S1; Filella and Rodríguez-Murillo 2017). Ga does not show many correlations. Finally, we found no strong correlations between TCEs and physicochemical parameters such as pH, Eh and temperature.

We also analysed the correlations between the variables. Fig. S2 shows the Tree diagram for 18 trace elements for which all the results of bottom sediment tests are taken, regardless of the sampling location. The combined results of all the Biała Przemsza River bottom sediments, presented in Table S3, show that Ge correlates very strongly with Te, and Tl correlates with As, Pb and Zn. Te correlated most strongly with Ni, V and Mn.

**Fig. 4:**
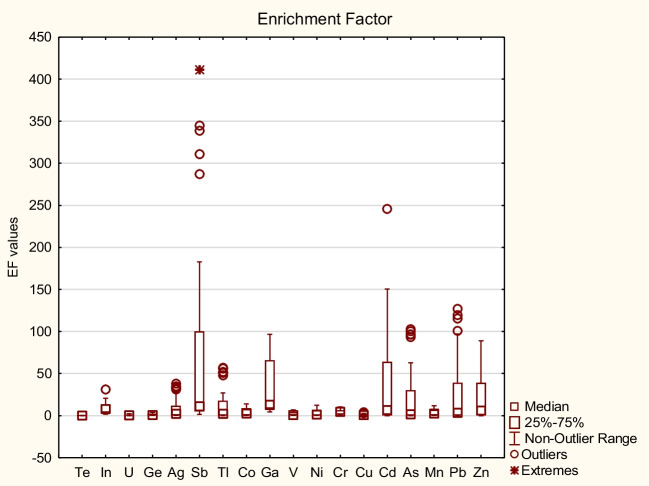
The box plot of the calculated *EF* (enrichment factor) of elements in the Biała Przemsza River bottom sediments

The *EF* values for heavy metals were calculated using Element Al as *MeRef* and in reference to the background values in the Biała Przemsza River sediments (Jabłońska-Czapla 2015; Lis and Pasieczna 1995), according to Eq. (5). As shown in Fig. 4, *EF* values of Sb, Tl, Ga, Cd, As, Pb and Zn exhibit severe extreme enrichment, which is mostly found in BP3 to BP5. However, no enrichment is observed in the sampling locations of BP1 and BP2, except for Sb, Ga and Cr. Additionally, In and Ag show moderate to significant enrichment (BP3 to BP5), whereas U and Te exhibit moderate to no enrichment in all sampling locations. Overall, excluding Ge, the *EF* results (Fig. 4) are consistent with other contamination indices such as *I*_*geo*_, *P*_*I*_ and *CF*, where all indices indicate no contamination signs concerning Ge except for *EF*, which shows minor to moderate enrichment from BP3 to BP5.

### Mobility of selected TCEs from the Biała Przemsza river bottom sediments

The negative impact of the presence of certain elements in the bottom sediments on the environment depends on their total concentration as well as their form, which determines their actual and potential instability and bioavailability. Therefore, knowledge of potentially toxic element speciation is crucial from the environmental risk point of view. A method commonly used to determine the fractionation of elements in the soil is sequential extraction.

**Fig. 5:**
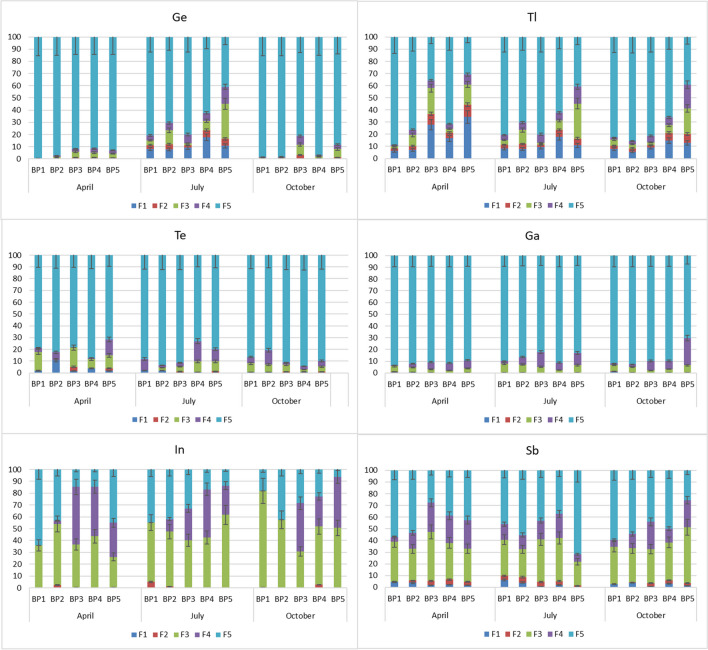
Sequential chemical extraction of selected technology-critical elements [%] in the Biała Przemsza River at various sampling points: BP1 — Chrząstowice, BP2 — Klucze; BP3 — Dąbrowa Górnicza Okradzionów; BP4 — Sławków, BP5 — Sosnowiec. Fractions: F1 — non-specifically sorbed elements; F2 – specifically sorbed elements; F3 – amorphous and weakly crystalline hydrated Fe and Al oxides; F4 – crystallised hydrated Fe and Al oxides; and F5 – elements related to silicates

Figure 5 shows the results of the sequential chemical extraction of the Biała Przemsza River bottom sediments. We fractionated the bottom sediments collected during three seasons: spring (April), summer (July) and autumn (October). We chose this approach to determine whether there are seasonal changes in the composition of bottom sediments. We employed the modified sequential extraction methodology described by Wenzel et al. (2001) to examine As and by Lewińska and Karczewska (2019) to examine Sb.

The Sb in bottom sediments was associated mainly with silicates (fraction F5) and amorphous oxides (hydroxides) of Mn and Fe (fraction F3). The share of Sb in fraction F3 was 20–48%, while the share of Sb in the residual fraction was 28–72%. Fraction F3 should also be considered potentially soluble because Sb can be mobilised under reducing conditions and such conditions prevailed in the Biała Przemsza River bottom sediments. Similarly, fraction F4 associated with crystalline oxides can be released in an oxygen-free environment. However, the Biała Przemsza River does not have anaerobic areas; hence, fraction F4 can be considered immobile. The smallest share of Sb occurred in the first two mobile fractions. We did not observe seasonal changes in Sb fractionation.

In the Biała Przemsza River bottom sediments, Ge was mainly associated with the residual fraction (40–99%). We made similar observations regarding the share of individual fractions of the bottom sediments collected in spring and autumn. During those seasons, the BP1 and BP2 bottom sediments contained strongly demobilised Ge in the residual fraction, while in the BP3–BP5 bottom sediments, the share of Ge in the mobile fractions increased slightly. The sequential chemical extraction of the bottom sediments collected in summer showed that Ge in the bottom sediments was largely associated with the mobile fractions, including fractions F1 (8–18%), F2 (2–6%) and F3 (4–29%) (Fig. 5). The share of Ge in the mobile fractions of the bottom sediments collected in July increased along the course of the river: the BP5 bottom sediments had the largest share of the mobile fractions.

The share of Tl in the mobile fractions of the bottom sediments increased along the course of the Biała Przemsza River. Figure 5 shows the level of variability of Tl in the bottom sediments. In spring at BP1 and BP2, Tl in the bottom sediments was mostly in a demobilised form, specifically in the residual fraction. However, the bottom sediments collected at the subsequent sampling points had a higher share of Tl in the mobile fractions; at BP5, the share of Tl in the mobile fractions was 40%. In summer, the share of Tl in the residual fraction (41–81%) decreased along the course of the river. Irrespective of the season, the BP5 bottom sediments had the highest share of Tl in the mobile fractions.

Tellurium in the bottom sediments was mainly associated with the residual immobile fraction (72–93%). Among the mobile fractions, we found Te mainly in fraction F3, with the maximum share of 16% measured at BP3 in April.

Similar to Te, Ga in the bottom sediments was mainly in the residual fraction. The maximum share of Ga in fraction F3 was only 7%. We found the largest share of Ga in fraction F4 (associated with well-crystallised, hydrated Fe and Al oxides) in the BP5 bottom sediments in October (23%).

Indium was found in the bottom sediments mainly in the form of amorphous and poorly crystallised Fe and Al hydroxy-oxides (fraction F4), which under favourable reducing conditions can release In into water. The share of In in fraction F3 was 25–81%.

The *RAC* calculated based on the sequential chemical extraction results showed that due to the way the elements are bound in the Biała Przemsza River bottom sediments, there is an average risk of contamination by As, Tl and Mn (Table S7).

### Identification of TCE sources

One of the most important factors related to the determination and distribution of elements in bottom sediments is their origin. Pearson’s correlation analysis (PCA) is frequently applied in this respect (Fang et al. 2019; Zhang et al. 2018). Here, PCA coupled with varimax rotation (factorial analysis) (Abdi and Williams 2010) was performed. PCA revealed that 76% of the total variance was caused by principal component 1 (PC1), while PC2 explained 14% of the variance. Importantly, all the elements except Cr had a strong correlation with PC1 (Fig. S4), which suggests there could be a unified source of these elements. Cr had a better correlation with PC2; thus, this element is more likely to be derived from a different source. After varimax rotation (Fig. S4), TCEs did not behave similarly. Sb and Tl along with some typical heavy metal pollutants — U, Cd, Cu, Ag, As, Pb and Zn — were significantly and positively correlated with Factor 1, which means these trace elements had a positive significant correlation with 53% of the total variance. On the other hand, Factor 2, which explained 37% of the total variance, was significantly and positively correlated with Te, Ge and V, as well as Cr, Ni, Mn and Co. While all the trace elements from Factor 2 showed a somewhat a moderate correlation with Factor 1, Cr did not show any correlation with Factor 1. For this reason, the geochemical behaviour of Cr was reflected differently in the analyses. Notably, while performing a varimax orthogonal rotation, the total variance remains the same (90% for both components or factors). However, varimax rotation redistributes the variance among the rotated components to create a simpler and more interpretable structure. This is achieved by boosting the high loadings and making the lower loadings less relevant.

The Kruskal–Wallis test was used to compare elemental mass fraction between sampling points. Before discussing the results, it is important to point out that in general, all the analysed elements had the lowest mean mass fractions at BP1 followed by BP2, except for Cr. Thus, BP1 could be considered a good reference for comparisons, followed by BP2. Except for Cr, the mass fractions of all elements at BP3 and BP5 were significantly different from the c mass fractions at BP1. Hence, the primary source of pollution is predicted to be from the locations. Obviously, for the Factor 1 elements, there were no significant differences between the mass fractions at BP3 and BP5, but there were significant differences between the mass fractions at BP3 and BP5 and BP2. Therefore, it is possible that the notable spike in the mass fractions of these elements occurred somewhere between BP2 and BP3 (Fig. S5). Unlike the Factor 1 elements, not all Factor 2 elements behaved similarly (Fig. S5). We found significant differences in the Mn, Co, Ni and Ge mass fractions when we compared BP1 and BP2 with BP3 and BP5. These data again indicate that BP3 and BP5 are potential pollution sources. When excluding BP1, there were no significant differences in the Ga and Te mass fractions between the sampling points. Finally, V behaved like Ga and Te, but there were also noticeable differences between BP4 and BP5.

Consistent results were obtained by performing HCA on the Spearman correlation matrix data. Various clustering possibilities emerged based on single linkage according to Euclidean distances (Fig. S2). Even though most elements, except for Cr and In, showed a high to very high positive correlation with each other, some exhibited stronger correlations than others. The Factor 1 elements had relatively strong correlations based on the easily formed clusters at relatively short Euclidean distances. On the other hand, the Factor 2 elements, including Ga, had higher Euclidean distances, indicating weaker correlations compared with the Factor 1 elements. Of note, Ni and Co showed very high correlations with most elements of both factors (Table S4). Additionally, there were strong correlations between In and the Factor 1 elements. Specifically, we only detected In at BP3–BP5, which suggests that this element is present in areas with a high *I*_*geo*_ that are heavily polluted. Lastly, we examined correlations between trace elements and physicochemical properties. There were no significant correlations between any of the trace elements and pH in the basic range. In addition, excluding In, all trace elements showed moderate to strong correlations with organic matter, where Pb, Ag and Ge exhibited moderate correlations, and V, Cr and Ni exhibited strong correlations. These data emphasise the crucial role of organic matter and its organic acids in forming complexing compounds with trace elements, thus affecting their retention and solubility. Moreover, with the exception of Ag, all Factor 1 elements and In displayed notably weak negative correlations with the redox potential. This indicates that increasing the reducing medium would slightly decrease the mass fraction of these elements.

Figure 1 shows the river and sampling points in addition to the mining activity areas. Notably, the east has Zn-Pb ore mines, while the west has coal mines. The different kinds of mining lands might be plausible anthropogenic source of pollution corresponding to the Factor 1 and 2 elements, resulting in a high spike of these elements particularly in areas located near BP3 and BP4.

The river catchment can be split into three main sections. BP1 and BP2 are in an area with minimal mining and other anthropogenic activities, resulting in low anomalies. In the second section, particularly at BP3, due to the high level of clay and organic matter that acts like an adsorbent, we can see the most significant impact of mining and human activities caused by Zn-Pb ore mines found in the Olkusz, Bukowno and Bolesław regions. This has led to the highest anomalies related to the element concentration. The most possible explanation is that the calamine soils and discharges in the vicinity of Zn-Pb ores (Swęd et al. 2022) are transported by erosion and flow towards the Biała Przemsza River via tributaries, which carry trace elements (Zn, Pb, Cd, As, Ag and Cu) and TCEs (Sb and Tl). Pasieczna and Lis (2008) conducted a study in the Olkusz region and made a similar assumption. Although Zn-Pb ores are natural sources of elements that cause aberrations, human activity has undoubtedly played a major role in exacerbating these abnormalities. Apart from trace elements from the Zn-Pb mining industry, wastewater discharge from various industrial sources – including Co, Cu, Li, Mn, Mg, Na, Ni, Rb and Sr — has also been reported. Finally, in the third section, specifically at BP5, Cr, Mn, Co, V, Ge, Ni and Te showed relatively higher anomalies. However, there is likely a combination of different sources of pollution. It is crucial to recognise that coal contains both mineral and organic components, and the organic part serves as a source of numerous primary and trace elements (Lewińska-Preis et al. 2021; Parzentny 2020), including those found in Zn-Pb ores. Furthermore, coal fly ash has the potential to transport and precipitate TCEs such as Ga, Ge and V and other chemicals into the river (Kursun Unver and Terzi 2018). Pasieczna et al. (2020) found in the Upper Silesian agglomeration that Co and V could potentially originate from a combination of geogenic-anthropogenic sources with a stronger influence of the geogenic source, while Cr and Ni could arise from the iron and steel industry. On the contrary, Kicińska and Wikar (2021) suggested that Ni and Cr might have originated from the application of phosphorus fertilisers and the use of municipal wastewater and sewage in agricultural soils from Sosnowiec and Bukowno. In addition, they pointed out the high potential for mobility of Zn, Ni and Cr in Bukowno and Pb in Sosnowiec. In China’s Hunan province, which is known for non-ferrous mining including Zn-Pb ores, researchers conducted extensive geochemical research to detect the impact of pollution sources on sediments, particularly in the Xiangjiang River and the Dongting Lake. Fang et al. (2019) found that the distribution of metals relevant to Zn-Pb ores in sediments varies spatially and is derived from both geogenic and anthropogenic processes in the Xiangjiang River. Similarly, the Biala Przemsza River exhibits a relatively heterogeneous distribution of these metals, despite the different proportions, which can be attributed to different weathering processes. Nevertheless, it is noteworthy that the distribution of trace elements, which have distinct physicochemical characteristics, might be influenced by the host material in which they are incorporated. For example, elements derived from Zn-Pb ores are generally hosted within Fe–Mn oxides and organic matter, while other trace metals of terrigenous sources may reside mostly in refractory material such as clay minerals (Fang et al. 2019, 2021, 2023).

### TCEs as indicators of anthropogenic contamination and assessment of the bottom sediment contamination

Several indices were calculated to estimate the pollution of the Biała Przemsza River, namely *I*_*geo*_, *P*_*I*_, *CF*, *EF* and the Sb/As ratio (Weissmannová and Pavlovský 2017).

**Fig. 6:**
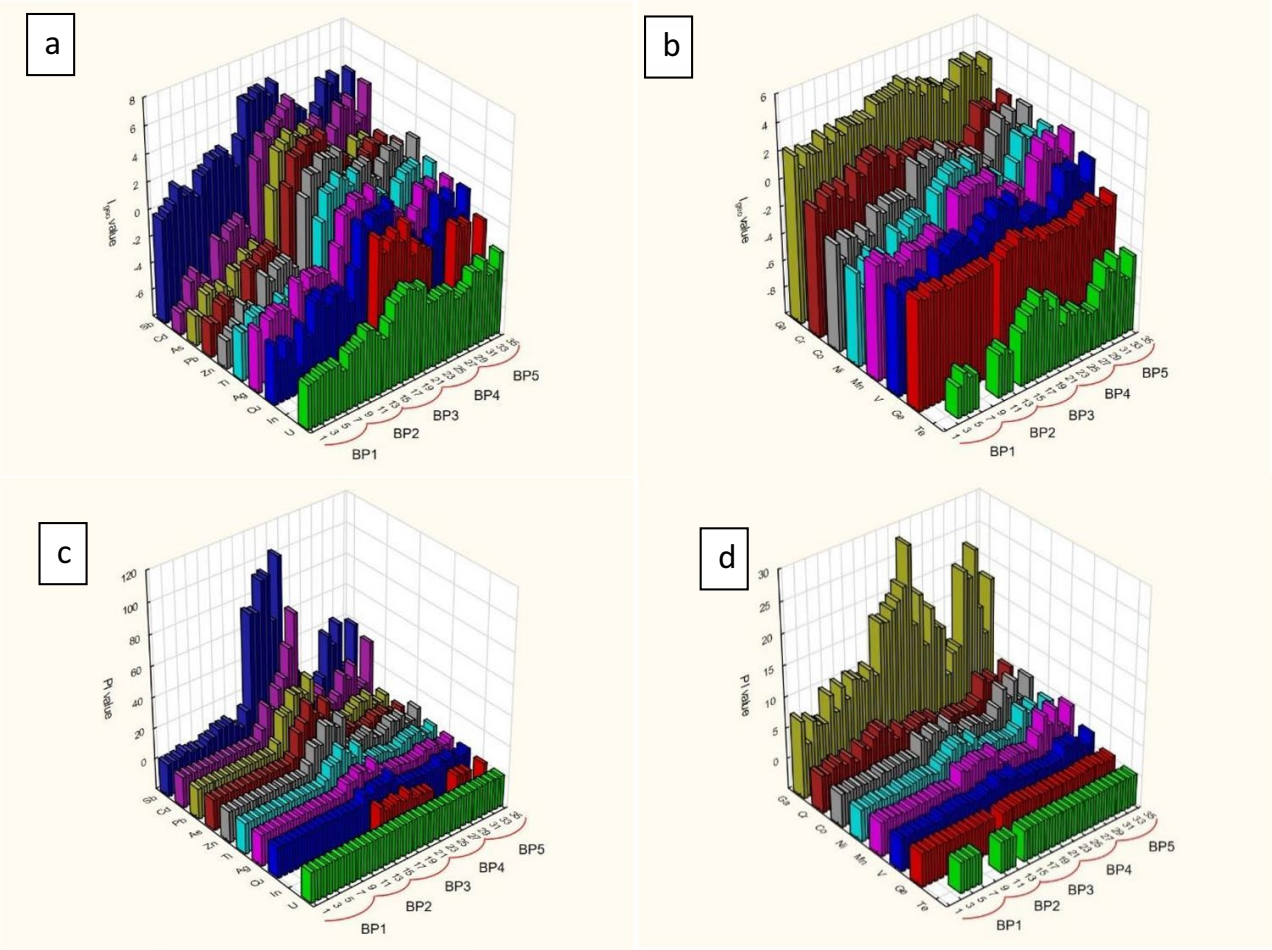
Spatial and temporal distributions of the geoaccumulation index (*I*_*geo*_): **a** represents elements connected to Factor 1 in PCA analyses and **b** represents elements connected to Factor 2 in PCA analysis and gallium; and the pollution index (*P*_*I*_): **c** represents elements connected to Factor 1 in PCA analyses and **d** represents elements connected to Factor 2 in PCA analysis and gallium. All the data in the graph are duly arranged to represent temporal and spatial changes in order

Figure 6 displays the spatial and temporal distribution of *I*_*geo*_ and *P*_*I*_ values for two major groups. The first group corresponds to Factor 1, and the second corresponds to Factor 2, in addition to Ga. It is worth noting that both *I*_*geo*_ and *P*_*I*_ indicate moderate to extreme levels of contamination for Sb, Cd, Pb, Zn, As, Tl and In, particularly in the BP3 sampling location. However, Co, Mn, Ni and V show moderate contamination in BP5 and moderately less contamination in BP3. Interestingly, Ga, like Sb, exhibits moderate to extreme contamination in all sampling locations, while Cr shows relative spikes in BP2 and BP5. It is noteworthy that except for Sb, Ga and Cr all elements’ *I*_*geo*_ and *P*_*I*_ values expressed uncontaminated levels in BP1 and BP2 locations. The *CF* values presented in Table S8 provide similar results and interpretations as those discussed for *I*_*geo*_ and *P*_*I*_. The *CF* shows moderate to very high contamination of the bottom sediments for Sb, Ga, Cd, Pb, Zn, As and Tl at BP3 to BP5. The highest values are observed at BP3. Excluding Sb, Ga and Cd, the rest of the analysed elements show low contamination of bottom sediments at BP1 and BP2 Table 2.

**Table 2:** Contamination factor (CF); sampling points BP1 — Chrząstowice; BP 2 — Klucze Osada; BP 3 — Dąbrowa Górnicza Okradzionów; BP 4 — Sławków; and BP 5 — Sosnowiec

Sampling point	Te	In	Ge	Sb	Tl	Co	Ga	V	Ni	Cr	Cu	Cd	As	Mn	Pb	Zn
BP1	0.01	N/A	0.40	2.38	0.09	0.30	6.57	0.23	0.19	1.26	0.09	0.05	0.05	0.45	0.05	0.06
BP2	0.01	N/A	0.40	6.88	0.15	0.77	10.00	0.71	0.54	2.97	0.35	0.35	0.15	0.45	0.28	0.25
BP3	0.05	4.39	0.63	73.85	11.71	3.12	20.00	1.48	2.64	2.22	6.32	33.95	22.12	2.71	26.17	19.52
BP4	0.01	1.32	0.46	15.90	2.46	1.22	12.32	0.51	0.74	0.89	1.03	9.47	4.00	0.70	5.58	5.96
BP5	0.06	1.49	0.68	26.74	4.00	3.80	15.84	1.68	2.61	3.28	2.41	15.63	3.34	3.62	5.40	6.89
BP1–BP5	0.03	2.68	0.52	25.15	3.68	1.84	12.95	0.92	1.34	2.13	2.04	11.89	5.93	1.59	7.49	6.54
Background value [mg/kg]	3.06	0.084	2.77	0.12	1.52	3.24	0.91	13.46	9.26	10.23	19.73	5.03	13.09	359.9	244.7	648.8
Source of the background value	Jabłońska-Czapla (2015)	Klein et al. (2022b)	Klein et al. (2022b)	Jabłońska-Czapla (2015)	Jabłońska-Czapla (2015)	Lis and Pasieczna (1995)	Jabłońska-Czapla (2015)	Lis and Pasieczna (1995)	Lis and Pasieczna (1995)	Lis and Pasieczna (1995)	Lis and Pasieczna (1995)	Lis and Pasieczna (1995)	Lis and Pasieczna (1995)	Lis and Pasieczna (1995)	Lis and Pasieczna (1995)	Lis and Pasieczna (1995)

*N*/*A* no data

As part of the study of the Biała Przemsza River bottom sediments, we determined the Sb/As ratio, which indicates the impact of mining and metallurgical industry emissions. The average value for all tested bottom sediment samples was 0.203, which is similar to the value obtained by Sharifi et al. (2016).

Based on pollution indices, the most heterogeneous elements (Factor 1), corresponding to Zn-Pb ores such as Sb, Cd, Tl and In, resulted in moderate to extreme contamination. This was particularly evident at BP3 near a mining area, where we clearly observed the spatial impact of the *I*_*geo*_ (see Fig. 6). On the other hand, the homogeneous group corresponding to Factor 2 elements, e.g. Ga, Co, V, Ge and Te showed a mostly uniform spatial distribution with some spikes and drops characterised by low to moderate contamination levels. It is important to note that the results based on the contamination indices differed slightly from PCA because these indices consider the influence of the background values, while PCA was conducted only with the mass fractions of the elements in the samples. For example, Zn and Pb have the highest mass fractions (17.40 and 9.35 g/kg, respectively), while Sb and Cd have the highest contamination levels based on the *I*_*geo*_ values (6.11 and 5.62, respectively).

The same holds true for the *P*_*I*_. We must add that while both Sb and Ga showed relatively higher consistency compared with the other analysed TCEs (with a *CF* of 25.15 and 12.94, respectively), there were clear differences in their mass fractions at BP3. On the other hand, Tl exhibited a similar spatial distribution as other Zn-Pb ore elements. In, however, was only present at BP3–BP5 and showed moderate contamination levels.

Based on TCE’s assessment, the pollution indices suggest that Sb and Ga are more subjected to increase causing higher levels of contamination than In, Co, V and Tl, which display relatively lower contamination levels. On the other hand, Ge and Te indicate no contamination in any of the measured samples, along with their uniform spatial and temporal distributions, as shown in Fig. 6. Apart from this, the heterogeneous group mostly indicates anthropogenic contamination, while the homogenous group may reflect geogenic contamination with possible limited interference.

The increasing consumption of TCEs has increased their concentrations in the environment (Jabłońska-Czapla et al. 2023). There has been research on the use of emerging contaminants as indicators of anthropogenic pollution for groundwater (Amiel et al. 2021) and surface water (Jabłońska-Czapla and Grygoyć 2022). In this paper, for the first time we tried to determine whether selected TCEs can serve as an indicator of pollution in bottom sediments. An increase in the concentration of an element, including TCEs, in the environment is influenced by geogenic and anthropogenic factors. Therefore, it is important to consider the Biała Przemsza River catchment area. The Biała Przemsza River flows through areas rich in Zn-Pb MVT-type stratoid deposits (Mikulski et al. 2020). We found a large variation in the metal and metalloid mass fractions in the Biała Przemsza River bottom sediments at each sampling point. In these areas, the Tl mass fractions in deposits (boreholes) were as high as 110 mg/kg, and the Ga mass fractions were as high as 177 mg/kg. In addition, Zn-Pb ore deposits are accompanied by elevated concentrations of Sb, which together with As are rarely found in deposits as sulphoarsenides (jordanite Pb_14_(As,Sb)_6_S_23_ and gratonite Pb_9_As_4_S_15_) (Mikulski et al. 2018). Therefore, at BP3 these TCEs could be indicators of anthropogenic pollution from the long exploitation of MVT deposits that has resulted in the transportation of these elements by erosion to the river. In a previous study, we showed that TCEs such as Te, Ge and In can be indicators of anthropogenic pollution of suspended matter and surface waters (Jabłońska-Czapla and Grygoyć 2022). In the Biała Przemsza River, In occurred mainly at BP3–BP5. The *P*_*I*_ (Håkanson 1980) showed that at BP3, In contamination was moderate to strong. Although its maximum mass fraction was 0.528 mg/kg, such amounts of In clearly indicate the anthropogenic nature of this contamination.

PCA showed that Te and Ge do not belong to the group of elements related to those associated with Zn-Pb ores (Fig. S4). On the other hand, similar to the suspended matter, the In mass fractions in the bottom sediments were so low that those could only be quantified in some of the samples, mainly from BP3–BP5. Favourable conditions such as an increase in pH can affect the release of TCEs. Such conditions may occur when the wastewater polluting the river water contain underground water from the mine (Jabłońska-Czapla et al. 2016).

Amiel et al. (2021) used yttrium (Y), rhodium (Rh), Ga, Ge and Tl as indicators of groundwater contamination due to their low background concentrations. In field studies, other TCEs with a low abundance could be used based on the specific environmental conditions and contamination sites. We showed that in the case of the Biała Przemsza River, Ge, Te and In can serve as indicators of anthropogenic contamination in bottom sediments. Klein et al. (2022b) drew similar conclusions after studying the Rhine River. They identified Ge and In as emerging contaminants, which indicates the anthropogenic nature of contamination with these TCEs in the river ecosystem.

## Conclusion

The analysis of selected TCEs and other elements in the Biała Przemsza River bottom sediments supports the following conclusions:

1. Low-concentration TCEs can identify human-caused contamination. Sb and Tl indicate Zn-Pb ore mining. Co, V, Ge and Te indicate coal and coal fly ash effluents. Although the current concentrations and contamination levels of Ge, In and Te are low and consistent in Biała Przemsza River, they are vulnerable to boost in the future due to the exponential increase of waste electrical and electronic equipment (WEEE).
2. The selected TCEs in the bottom sediments are characterised into two major groups, the first is the heterogeneous group that originated from the Zn-Pb ores including Sb and Tl, which spike mainly in BP3. The second is the homogenous group such as Ga, Co, V, Ge and Te denoted by relatively low and consistent concentrations that rise in BP5.
3. All four contamination indices (*I*_*geo*_, *P*_*I*_, *CF* and *EF*) show that bottom sediments of the Biała Przemsza River are highly contaminated by Sb, Ga, Tl, Cd, As, Zn and Pb, mainly in BP3. Additionally, Co, In and V exhibit moderate contamination, especially in BP3 and BP5, according to all indices except for *EF*. However, Ge and Te reveal a low degree of contamination in all sampling locations.
4. Based on the analysis of PCA, HCA and the spatial distribution for *I*_*geo*_ and *P*_*I*_ contamination indices, it can be concluded that the heterogeneous group shows signs of anthropogenic contamination, specifically with Sb and Tl, as well as indium. On the other hand, the homogenous group may indicate geogenic contamination with limited interference from elements, e.g. Ga, Co and V.
5. The proportion of selected TCEs in the mobile fraction of the bottom sediments changes depending on the season. Out of the TCEs that were tested, Sb and In were the most mobile, regardless of where or when the samples were taken. Tl showed high mobility from the bottom sediments, especially at BP3–BP5. This means that it is more available, even though it may be present at lower concentrations. On the other hand, Te and Ga were less mobile in the sediments, although they were not completely immobile. Based on the sequential chemical extraction, the *RAC* calculation revealed that the way the elements are bound in the Biała Przemsza River bottom sediments resulted in an average risk of contamination by As, Tl and Mn.

TCEs are rarely evaluated in the environment (Gil-Díaz et al. 2019, 2020; Jabłońska-Czapla and Grygoyć 2021; Ospina-Alvarez et al. 2014), and so far there are no defined limit values or maximum permissible concentrations of these elements in water or bottom sediments. There is also a lack of reference values to assess environmental contamination by TCEs. Only recently the scientific community started to expand research on this topic and to compare results (Klein et al. 2022a, 2022b). There needs to be mutual cooperation and comparison of the obtained research results to expand knowledge on TCEs (Filella 2020). The use of TCEs will increase considerably over the next decades. Therefore, it is necessary to extend the research on the occurrence of TCEs in the environment and to learn about their transformations also in terms of ecotoxicological aspects. This investigation enhances our understanding of TCEs’ environmental dynamics, providing valuable insights into their potential utilisation as reliable indicators of human activities. The findings contribute to sustainable resource management and environmental conservation discussions, emphasising the need for mitigating the environmental impact of TCE-related industries.

### Supplementary Information

Below is the link to the electronic supplementary material.

Supplementary file1 (DOCX 418 KB)

## Data Availability

Not applicable.

## Sample availability

Samples of the compounds are available from the authors.
