# Supplementary material for: Assessment of contamination, mobility and application of selected technology-critical elements as indicators of anthropogenic pollution of bottom sediments
Source: Environ Sci Pollut Res Int. 2024 Jul 30;31(37):49694–714. doi: 10.1007/s11356-024-34377-5 (PMC11324682; doi:10.1007/s11356-024-34377-5)
Supplement: Supplementary file 1 — Supplementary file1 (DOCX 418 KB) [file 11356_2024_34377_MOESM1_ESM.docx]

Assessment of contamination, mobility and application of selected technology-critical elements as indicators of anthropogenic pollution of bottom sediments

Magdalena Jabłońska-Czapla, Katarzyna Grygoyć, George Yandem

^1^Institute of Environmental Engineering of Polish Academy of Sciences, M. Sklodowskiej-Curie 34 St., 41-819 Zabrze, Poland

Phone +48 32 271 64 81

Fax +48 32 271 74 70

*Corresponding author: magdalena.czapla@ipispan.edu.pl

**Table S1.** Basic validation parameters and recovery of the stream sediment certified reference material.

| Analyte | Isotope | LOQ [mg/kg] | CRM NCS DC 79309  from certificate  [mg/kg] | CRM NCS DC 79309  measured  [mg/kg] | Recovery  [%] | Uncertainty  [%] |
| --- | --- | --- | --- | --- | --- | --- |
| Te | 126 | 0.02 | 0.4 ± 0.1 | 0.414 ± 0.054 | 103 | 13 |
| In | 115 | 0.05 | 1.9 ± 0.3 | 1.82 ± 0.24 | 96 | 13 |
| U | 238 | 0.1 | 7.8 ± 0.9 | 9.4 ± 1.1 | 120 | 12 |
| Ge | 74 | 0.2 | 1.81 ± 0.21 | 1.85 ± 0.30 | 102 | 16 |
| Ag | 107 | 0.1 | 3.2 ± 0.4 | 3.37 ± 0.51 | 106 | 14 |
| Sb | 121 | 0.08 | 14.9 ± 1.2 | 15.5 ± 2.2 | 104 | 14 |
| Tl | 205 | 0.05 | 2.9 ± 0.4 | 3.11 ± 0.47 | 107 | 15 |
| Co | 59 | 0.05 | 8.5 ± 0.8 | 7.9 ± 1.1 | 93 | 14 |
| Ga | 69 | 0.05 | 18.5 ± 0.9 | 21.4 ± 2.1 | 116 | 10 |
| V | 51 | 0.05 | 47 ± 3 | 45.1 ± 3.8 | 96 | 8.5 |
| Ni | 60 | 0.08 | 14.3 ± 1.0 | 14.1 ± 2.0 | 99 | 14 |
| Cr | 53 | 0.08 | 40 ± 3 | 39.7 ± 6.7 | 99 | 17 |
| Cu | 65 | 0.08 | 79 ± 3 | 74.4 ± 9.7 | 94 | 13 |
| Cd | 114 | 0.02 | 2.3 ± 0.2 | 2.14 ± 0.20 | 93 | 9.6 |
| As | 75 | 0.1 | 188 ± 13 | 167 ± 27 | 89 | 16 |
| Mn | 55 | 0.2 | 2490 ± 84 | 2250 ± 292 | 90 | 13 |
| Pb | 208 | 0.08 | 636 ± 22 | 615 ± 61 | 97 | 9.8 |
| Zn | 66 | 0.1 | 373 ± 14 | 352 ± 84 | 94 | 24 |

LOQ – Limit of Quantification, CRM – Certified Reference Material, LOQ=3*LOD

**Table S2.** Sequential extraction procedure based on Wenzel et al. (2001).

| **Fraction** | **Extraction solution** | **Extraction conditions** | **Sample to solution ratio (SSR) [ml]** | **Rinsing** |
| --- | --- | --- | --- | --- |
| 1 | 0.05 M ammonium sulfate (NH_4_)_2_SO_4_ | 4 hours of shaking, temperature 20℃ | 1:25 | - |
| 2 | 0.05 M ammonium dihydrogen phosphate (NH_4_)H_2_PO_4_ | 16 hours of shaking, temperature 20℃ | 1:25 | - |
| 3 | 0.2 M NH_4_- oxalate buffer,  pH 3.25 | 4 hours of shaking in darkness, temperature 20℃ | 1:25 | 0.2 M NH_4_- oxalate buffer, pH 3.25; SSR: 1:12,5; 10 minutes of shaking in darkness |
| 4 | 0.2 M NH_4_- oxalate buffer,  0.1 M ascorbic acid  pH 3.25 | 30 minutes in a water bath at temperature 96±3℃ | 1:25 | 0.2 M NH_4_- oxalate buffer, pH 3.25; SSR: 1:12,5; 10 minutes of shaking in darkness |
| 5 | 10 ml HNO_3_, 4ml H_2_O_2_, 4ml HF | Microwave digestion, 190℃,  50 minutes | 1:50 | - |

**Table S3.** The basic descriptive statistics.

| Variable | Descriptive Statistics | | | | | | |
| --- | --- | --- | --- | --- | --- | --- | --- |
|  | \| Valid N \| \| --- \| | \| Mean \| \| --- \| | \| Confidence interval limits \| \| --- \|  \| From low to high limit \| \| --- \| | | \| Minimum \| \| --- \| | \| Maximum \| \| --- \| | \| SD \| \| --- \| |
| \| Te \| \| --- \| | 28 | 0.104 | 0.071 | 0.136 | 0.02254 | 0.29 | 0.084 |
| \| In \| \| --- \| | 14 | 0.225 | 0.137 | 0.314 | 0.08706 | 0.53 | 0.153 |
| \| U \| \| --- \| | 35 | 1.092 | 0.819 | 1.366 | 0.24280 | 3.26 | 0.796 |
| \| Ge \| \| --- \| | 34 | 1.432 | 1.290 | 1.575 | 0.93664 | 2.46 | 0.409 |
| \| Ag \| \| --- \| | 34 | 2.122 | 1.227 | 3.016 | 0.09509 | 9.51 | 2.563 |
| \| Sb \| \| --- \| | 35 | 3.028 | 1.792 | 4.265 | 0.15460 | 12.50 | 3.600 |
| \| Tl \| \| --- \| | 35 | 5.600 | 3.045 | 8.155 | 0.08604 | 27.71 | 7.437 |
| \| Co \| \| --- \| | 35 | 5.978 | 4.222 | 7.735 | 0.49357 | 19.23 | 5.112 |
| \| Ga \| \| --- \| | 35 | 11.750 | 9.871 | 13.629 | 3.29878 | 25.59 | 5.471 |
| \| V \| \| --- \| | 35 | 12.401 | 9.103 | 15.699 | 1.82265 | 32.68 | 9.601 |
| \| Ni \| \| --- \| | 35 | 12.435 | 8.447 | 16.423 | 0.67606 | 36.72 | 11.609 |
| \| Cr \| \| --- \| | 35 | 21.738 | 17.356 | 26.120 | 6.31390 | 49.39 | 12.756 |
| \| Cu \| \| --- \| | 35 | 40.226 | 21.771 | 58.680 | 0.43265 | 206.39 | 53.722 |
| \| Cd \| \| --- \| | 35 | 59.770 | 31.157 | 88.383 | 0.09165 | 371.98 | 83.296 |
| \| As \| \| --- \| | 35 | 77.666 | 35.227 | 120.105 | 0.31235 | 434.93 | 123.544 |
| \| Mn \| \| --- \| | 35 | 570.553 | 384.866 | 756.240 | 76.60656 | 1964.73 | 540.555 |
| \| Pb \| \| --- \| | 35 | 1834.236 | 917.971 | 2750.501 | 7.41401 | 9347.92 | 2667.345 |
| \| Zn \| \| --- \| | 35 | 4239.869 | 2449.182 | 6030.557 | 16.42094 | 17394.73 | 5212.881 |

**Table S4.** Spearman correlation matrix of all results of the Biała Przemsza River bottom sediments. Eh – redox potential; OM – organic matter

| Variable | Spearman Rank Order Correlations (data BP) MD pairwise deleted Marked correlations are significant at p <.05000 | | | | | | | | | | | | | | | | | | | | |
| --- | --- | --- | --- | --- | --- | --- | --- | --- | --- | --- | --- | --- | --- | --- | --- | --- | --- | --- | --- | --- | --- |
|  | \| Te \| \| --- \| | \| In \| \| --- \| | \| U \| \| --- \| | \| Ge \| \| --- \| | \| Ag \| \| --- \| | \| Sb \| \| --- \| | \| Tl \| \| --- \| | \| Co \| \| --- \| | \| Ga \| \| --- \| | \| V \| \| --- \| | \| Ni \| \| --- \| | \| Cr \| \| --- \| | \| Cu \| \| --- \| | \| Cd \| \| --- \| | \| As \| \| --- \| | \| Mn \| \| --- \| | \| Pb \| \| --- \| | \| Zn \| \| --- \| | \| Eh \| \| --- \| | \| pH \| \| --- \| | \| OM \| \| --- \| |
| \| Te \| \| --- \| | 1.00 |  |  |  |  |  |  |  |  |  |  |  |  |  |  |  |  |  |  |  |  |
| \| In \| \| --- \| | 0.34 | 1.00 |  |  |  |  |  |  |  |  |  |  |  |  |  |  |  |  |  |  |  |
| \| U \| \| --- \| | 0.84 | 0.71 | 1.00 |  |  |  |  |  |  |  |  |  |  |  |  |  |  |  |  |  |  |
| \| Ge \| \| --- \| | 0.93 | 0.29 | 0.77 | 1.00 |  |  |  |  |  |  |  |  |  |  |  |  |  |  |  |  |  |
| \| Ag \| \| --- \| | 0.86 | 0.84 | 0.93 | 0.81 | 1.00 |  |  |  |  |  |  |  |  |  |  |  |  |  |  |  |  |
| \| Sb \| \| --- \| | 0.87 | 0.89 | 0.96 | 0.81 | 0.98 | 1.00 |  |  |  |  |  |  |  |  |  |  |  |  |  |  |  |
| \| Tl \| \| --- \| | 0.87 | 0.82 | 0.94 | 0.85 | 0.98 | 0.97 | 1.00 |  |  |  |  |  |  |  |  |  |  |  |  |  |  |
| \| Co \| \| --- \| | 0.92 | 0.09 | 0.88 | 0.83 | 0.89 | 0.91 | 0.90 | 1.00 |  |  |  |  |  |  |  |  |  |  |  |  |  |
| \| Ga \| \| --- \| | 0.76 | 0.20 | 0.85 | 0.68 | 0.84 | 0.88 | 0.82 | 0.88 | 1.00 |  |  |  |  |  |  |  |  |  |  |  |  |
| \| V \| \| --- \| | 0.89 | 0.14 | 0.88 | 0.72 | 0.79 | 0.84 | 0.80 | 0.90 | 0.82 | 1.00 |  |  |  |  |  |  |  |  |  |  |  |
| \| Ni \| \| --- \| | 0.96 | 0.25 | 0.92 | 0.86 | 0.92 | 0.94 | 0.93 | 0.98 | 0.87 | 0.92 | 1.00 |  |  |  |  |  |  |  |  |  |  |
| \| Cr \| \| --- \| | 0.56 | 0.10 | 0.49 | 0.31 | 0.28 | 0.41 | 0.33 | 0.54 | 0.51 | 0.77 | 0.55 | 1.00 |  |  |  |  |  |  |  |  |  |
| \| Cu \| \| --- \| | 0.87 | 0.89 | 0.93 | 0.80 | 0.96 | 0.97 | 0.94 | 0.90 | 0.88 | 0.84 | 0.93 | 0.39 | 1.00 |  |  |  |  |  |  |  |  |
| \| Cd \| \| --- \| | 0.84 | 0.64 | 0.94 | 0.83 | 0.98 | 0.97 | 0.97 | 0.91 | 0.85 | 0.82 | 0.94 | 0.35 | 0.96 | 1.00 |  |  |  |  |  |  |  |
| \| As \| \| --- \| | 0.76 | 0.76 | 0.92 | 0.77 | 0.98 | 0.96 | 0.97 | 0.86 | 0.83 | 0.74 | 0.89 | 0.23 | 0.94 | 0.96 | 1.00 |  |  |  |  |  |  |
| \| Mn \| \| --- \| | 0.93 | 0.13 | 0.80 | 0.82 | 0.82 | 0.82 | 0.83 | 0.88 | 0.76 | 0.80 | 0.89 | 0.45 | 0.81 | 0.82 | 0.75 | 1.00 |  |  |  |  |  |
| \| Pb \| \| --- \| | 0.78 | 0.83 | 0.92 | 0.78 | 0.98 | 0.97 | 0.97 | 0.87 | 0.84 | 0.75 | 0.89 | 0.26 | 0.95 | 0.96 | 0.99 | 0.76 | 1.00 |  |  |  |  |
| \| Zn \| \| --- \| | 0.82 | 0.79 | 0.95 | 0.81 | 0.99 | 0.97 | 0.98 | 0.89 | 0.84 | 0.80 | 0.92 | 0.32 | 0.95 | 0.99 | 0.98 | 0.80 | 0.97 | 1.00 |  |  |  |
| \| Eh \| \| --- \| | -0.17 | -0.65 | -0.42 | -0.31 | -0.33 | -0.43 | -0.38 | -0.28 | -0.24 | -0.27 | -0.32 | -0.23 | -0.37 | -0.39 | -0.39 | -0.10 | -0.39 | -0.40 | 1.00 |  |  |
| \| pH \| \| --- \| | 0.15 | -0.11 | -0.12 | -0.05 | -0.05 | -0.01 | -0.06 | 0.11 | -0.02 | 0.07 | 0.07 | 0.15 | 0.02 | -0.01 | -0.10 | 0.08 | -0.08 | -0.05 | -0.05 | 1.00 |  |
| \| OM \| \| --- \| | 0.69 | 0.08 | 0.69 | 0.56 | 0.55 | 0.65 | 0.57 | 0.77 | 0.69 | 0.89 | 0.78 | 0.86 | 0.66 | 0.62 | 0.50 | 0.63 | 0.53 | 0.58 | -0.31 | 0.22 | 1.00 |

**Table S5.** Basic physicochemical parameters with the Clark coefficient in the bottom sediments of the Biała Przemsza River at various sampling points: BP1 – Chrząstowice, BP2 – Klucze; BP3 – Dąbrowa Górnicza Okradzionów; BP4 – Sławków, BP5 – Sosnowiec.

| **Data** | **Sampling point** | **pH** | **Eh [mV]** | **rH** |
| --- | --- | --- | --- | --- |
| **April** | **BP 1** | 7.47 | 239.8 | 23 |
|  | **BP 2** | 7.77 | -126.6 | 11 |
|  | **BP 3** | 7.65 | -210.7 | 8 |
|  | **BP 4** | 7.61 | -97.4 | 12 |
|  | **BP 5** | 7.77 | -222.6 | 8 |
| **May** | **BP 1** | 7.48 | -31.7 | 14 |
|  | **BP 2** | 7.72 | -243.1 | 7 |
|  | **BP 3** | 7.42 | -157.7 | 10 |
|  | **BP 4** | 7.6 | -303.5 | 5 |
|  | **BP 5** | 7.65 | -75.7 | 13 |
| **June** | **BP 1** | 7.49 | -109.4 | 11 |
|  | **BP 2** | 7.76 | -159.3 | 10 |
|  | **BP 3** | 7.37 | -178.3 | 9 |
|  | **BP 4** | 7.65 | -187.3 | 9 |
|  | **BP 5** | 7.63 | -119.3 | 11 |
| **July** | **BP 1** | 7.41 | -212.8 | 8 |
|  | **BP 2** | 7.49 | -251.2 | 7 |
|  | **BP 3** | 7.29 | -349 | 3 |
|  | **BP 4** | 6.98 | -93.9 | 11 |
|  | **BP 5** | 7.52 | -139.3 | 10 |
| **August** | **BP 1** | 7.56 | -185.4 | 9 |
|  | **BP 2** | 7.24 | -181.9 | 8 |
|  | **BP 3** | 7.44 | -298.3 | 5 |
|  | **BP 4** | 7.61 | -254.4 | 7 |
|  | **BP 5** | 7.63 | -117.7 | 11 |
| **September** | **BP 1** | 7.33 | -107.9 | 11 |
|  | **BP 2** | 7.55 | -292.3 | 5 |
|  | **BP 3** | 7.41 | -252.2 | 6 |
|  | **BP 4** | 7.35 | -136.5 | 10 |
|  | **BP 5** | 7.66 | -184.9 | 9 |
| **October** | **BP 1** | 7.71 | 34.7 | 17 |
|  | **BP 2** | 7.53 | -208.9 | 8 |
|  | **BP 3** | 7.75 | -309.5 | 5 |
|  | **BP 4** | 7.55 | -136.8 | 11 |
|  | **BP 5** | 7.69 | -168.7 | 10 |

Clark's coefficient rH = (Eh+0.068*pH)/0.03; Eh redox potential

**Table S6.** Granulometric analysis of the bottom sediments of the Biała Przemsza River at various sampling points: BP1 – Chrząstowice; BP 2 – Klucze Osada; BP 3 – Dąbrowa Górnicza Okradzionów; BP 4 – Sławków; BP 5 – Sosnowiec.

| Season | Sampling point | >2 mm  gravel fraction* | 2-1 mm  very coarse sand fraction | 1-0.5 mm  coarse sand fraction | 0.5-0.2 mm  medium sand fraction | 0.2-0.1 mm  fine sand fraction | <0.1 mm  dust and clay fraction | Unit |
| --- | --- | --- | --- | --- | --- | --- | --- | --- |
| Spring | BP 1 | 0.0 | 0.1 | 7.3 | 86.0 | 6.1 | 0.5 | % |
|  | BP 2 | 0.2 | 0.8 | 8.2 | 74.3 | 11.8 | 4.7 | % |
|  | BP 3 | 0.8 | 1.0 | 1.0 | 7.1 | 18.3 | 71.7 | % |
|  | BP 4 | 0.1 | 0.1 | 0.1 | 54.2 | 31.7 | 13.8 | % |
|  | BP 5 | 0.7 | 0.6 | 3.1 | 72.4 | 14.3 | 9.0 | % |
| Summer | BP 1 | 0.5 | 0.3 | 10.0 | 67.7 | 16.2 | 5.3 | % |
|  | BP 2 | 0.3 | 1.6 | 8.4 | 58.3 | 17.5 | 13.9 | % |
|  | BP 3 | 0.0 | 0.1 | 0.6 | 8.7 | 16.3 | 74.2 | % |
|  | BP 4 | 0.3 | 0.3 | 0.2 | 41.8 | 38.6 | 18.9 | % |
|  | BP 5 | 3.0 | 6.3 | 10.8 | 49.7 | 15.5 | 14.7 | % |
| Autumn | BP 1 | 0.1 | 0.5 | 29.2 | 66.8 | 2.5 | 0.9 | % |
|  | BP 2 | 0.5 | 0.7 | 9.8 | 72.5 | 13.0 | 3.4 | % |
|  | BP 3 | 0.0 | 0.2 | 1.0 | 14.5 | 22.6 | 61.7 | % |
|  | BP 4 | 0.3 | 0.2 | 0.4 | 63.7 | 28.4 | 7.0 | % |
|  | BP 5 | 3.9 | 2.7 | 20.8 | 52.4 | 12.1 | 8.0 | % |

* According to the Classification of grain size distribution of soils and mineral formations of the Polish Society of Soil Science of 2008 and PN-EN ISO 17892-4:2017-01

**Table S7.** Risk Assessment Code (RAC) values for the Biała Przemsza River (Perin et al., 1985).

| RAC | Values (%) | RAC for the Biała Przemsza River |
| --- | --- | --- |
| No risk | < 1 | Ag, Cr, Ge, In, Ga,Pb |
| Low risk | 1–10 | Co, Ni, Cu, Cd,V, Te, Sb,Zn, Rb, Sr, Ba |
| Medium risk | 11–30 | As, Tl, Mn |
| High risk | 31–50 | - |
| Very high risk | > 50 | - |

**Fig. S1.** Relationship between the occurrence of elements such as Zn, Cu or Pb and TCEs (Filella and Rodríguez-Murillo 2017)

**
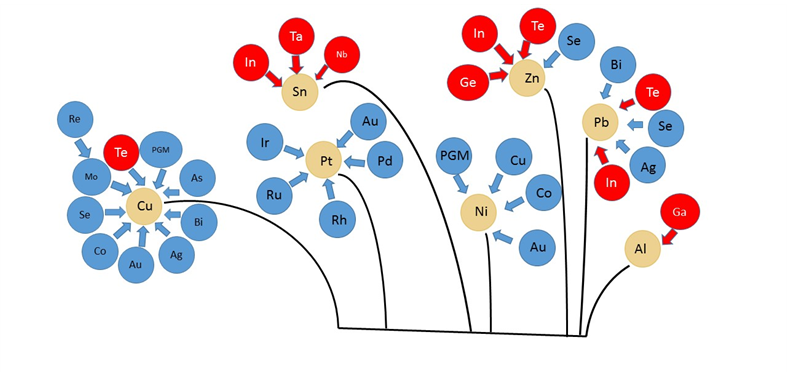
**

**Fig. S2.** Tree diagram for 18 trace elements (single linkage according to Euclidean distances).

**Fig. S3.** Physicochemical parameters – redox potential (Eh), temperature [°C] and pH – in the bottom sediments of the Biała Przemsza River at various sampling points: BP1 – Chrząstowice, BP2 – Klucze; BP3 – Dąbrowa Górnicza Okradzionów; BP4 – Sławków, BP5 – Sosnowiec.


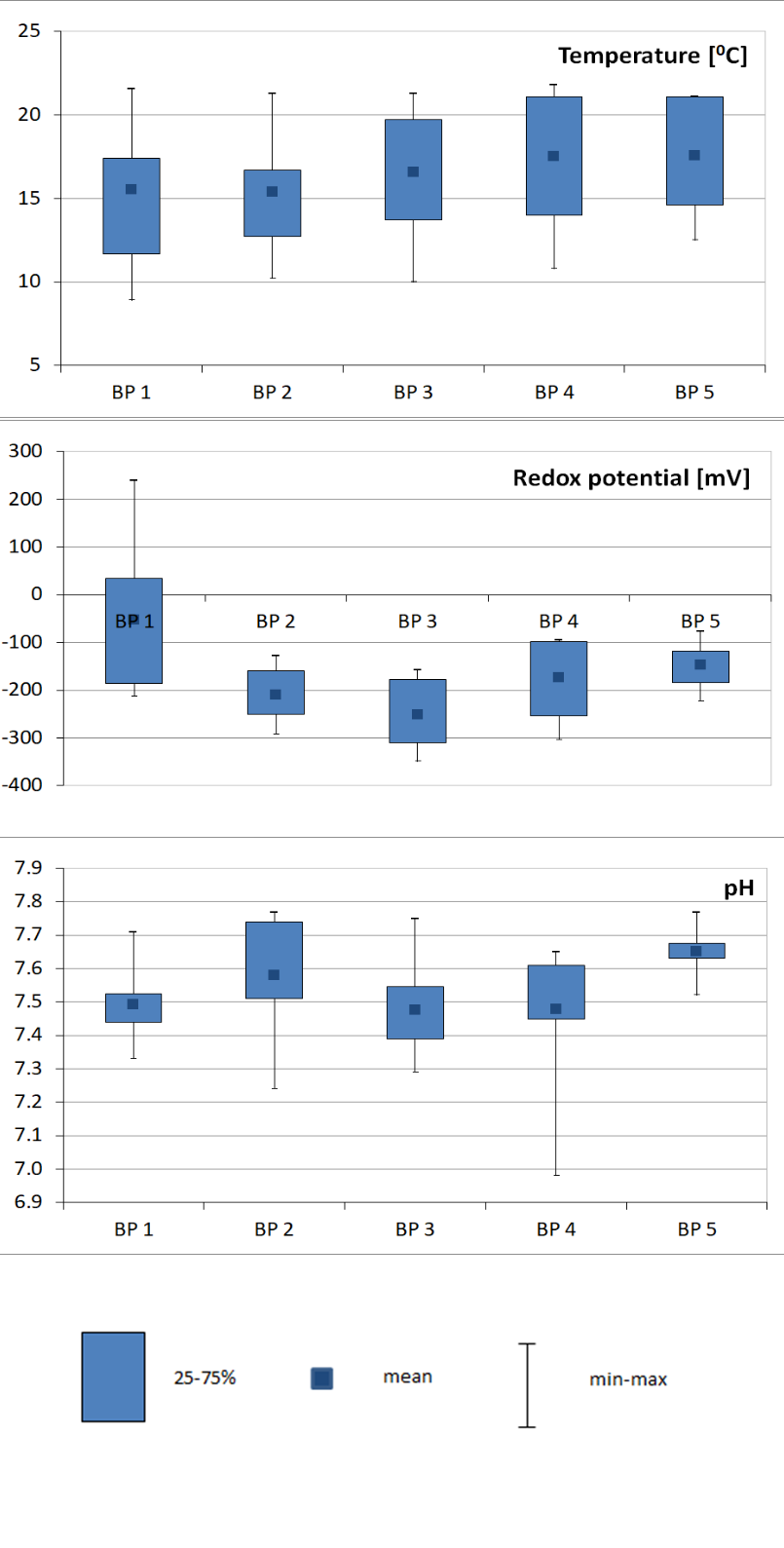


**Fig. S4.** a) Principal component analysis loading scatterplot (principal component 1 [PC1] vs PC2); b) Factor loadings (Factor 1 vs Factor 2) after varimax normalised rotation and principal component extraction.

a) b)

**Fig. S5.** Confidence intervals for the total concentrations
